# Targeting Immunoproteasome in Polarized Macrophages Ameliorates Experimental Emphysema Via Activating NRF1/2‐P62 Axis and Suppressing IRF4 Transcription

**DOI:** 10.1002/advs.202405318

**Published:** 2024-10-02

**Authors:** Bingxin Guo, Xing Shi, Qiong Jiang, Yuanwei Pan, Yuqiong Yang, Yuanyuan Liu, Shuyu Chen, Wenjiao Zhu, Laibin Ren, Ruifang Liang, Xue Chen, Haizhao Xu, Laiyou Wei, Yongjian Lin, Jinyong Wang, Chen Qiu, Haibo Zhou, Lang Rao, Lingwei Wang, Rongchang Chen, Shanze Chen

**Affiliations:** ^1^ Department of Pulmonary and Critical Care Medicine, Shenzhen Institute of Respiratory Diseases The First Affiliated Hospital (Shenzhen People's Hospital) and School of Medicine, Southern University of Science and Technology Shenzhen 518055 China; ^2^ Institute of Chemical Biology Shenzhen Bay Laboratory Shenzhen 518132 China; ^3^ National Clinical Research Center for Respiratory Disease, Guangzhou Institute of Respiratory Health, State Key Laboratory of Respiratory Disease First Affiliated Hospital of Guangzhou Medical University Guangzhou 510150 China; ^4^ Department of Respiratory Diseases and Critic Care Unit, Shenzhen Institute of Respiratory Disease, Shenzhen Key Laboratory of Respiratory Disease, Post‐doctoral Scientific Research Station of Basic Medicine, The Second Clinical Medical College Jinan University Guangzhou 510632 China; ^5^ College of Pharmacy Jinan University Guangzhou Guangdong 510632 China

**Keywords:** emphysema, immunoproteasome, IRF4, macrophage polarization, NRF1/2‐P62

## Abstract

Chronic obstructive pulmonary disease (COPD) stands as the prevailing chronic airway ailment, characterized by chronic bronchitis and emphysema. Current medications fall short in treatment of these diseases, underscoring the urgent need for effective therapy. Prior research indicated immunoproteasome inhibition alleviated various inflammatory diseases by modulating immune cell functions. However, its therapeutic potential in COPD remains largely unexplored. Here, an elevated expression of immunoproteasome subunits LMP2 and LMP7 in the macrophages isolated from mouse with LPS/Elastase‐induced emphysema and polarized macrophages in vitro is observed. Subsequently, intranasal administration of the immunoproteasome‐specific inhibitor ONX‐0914 significantly mitigated COPD‐associated airway inflammation and improved lung function in mice by suppressing macrophage polarization. Additionally, ONX‐0914 capsulated in PLGA nanoparticles exhibited more pronounced therapeutic effect on COPD than naked ONX‐0914 by targeting immunoproteasome in polarized macrophages. Mechanistically, ONX‐0914 activated autophagy and endoplasmic reticulum (ER) stress are not attribute to the ONX‐0914 mediated suppression of macrophage polarization. Intriguingly, ONX‐0914 inhibited M1 polarization through the nuclear factor erythroid 2‐related factor‐1 (NRF1) and NRF2‐P62 axis, while the suppression of M2 polarization is regulated by inhibiting the transcription of interferon regulatory factor 4 (IRF4). In summary, the findings suggest that targeting immunoproteasome in macrophages holds promise as a therapeutic strategy for COPD.

## Graphical abstract

This study performs immunoproteasome inhibitor ONX‐0194 effectively prevents the development of emphysema by modulating macrophage activation. Mechanistically, ONX‐0194 mitigates macrophage inflammation, involving the activation of the NRF1 and NRF2‐P62 axis in M1 polarization, and regulation of IRF4 in M2 polarization. These findings strongly suggest that targeting the immunoproteasome in macrophages holds promise as a therapeutic approach for COPD.

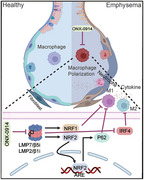

## 1 Introduction

Chronic obstructive pulmonary disease (COPD), the most prevalent chronic airway ailment, stands as the third leading cause of death globally. It is characterized by a blend of airway inflammation, obstructive bronchiolitis, airway remodeling, ultimately leads to emphysema and persistent airflow limitation.^[^ ^1^, ^2^ ^]^ Cigarette smoking serves as the primary etiological factor for COPD. However, even after smoking cessation, the airway inflammatory response can persist over time.^[^ ^3^ ^]^ Although inhaled corticosteroids (ICS) and long‐acting beta (2)‐agonists (LABAs) represent two well‐established treatments for COPD patients, they are palliative in nature. These treatments can only slow or halt disease progression and cannot restore or reverse the disease, especially in patients with emphysema.^[^ ^4^ ^]^ The World Health Organization (WHO) predicts that by 2030, COPD will affect nearly 400 million people worldwide.^[^ ^5^ ^]^ Hence, there exists an urgent need to develop innovative therapeutic approaches.

Alveolar macrophages (AMs), a specialized subset of lung resident phagocytes, play a pivotal role in the intricate landscape of COPD pathogenesis.^[^ ^3^, ^6^ ^]^ They exhibit remarkable adaptability, responding dynamically to stimuli such as cigarette smoke and microbial agents, thereby serving as vital conductors of inflammation in COPD.^[^ ^7^, ^8^ ^]^ Macrophages, inherently versatile, adopt two principal activation states, profoundly shaping the inflammatory milieu in the disease. One of these states involves the classical M1 macrophage activation pattern, associated with the Th1 immune response. Triggered by IFN‐γ/LPS, M1 macrophages produce significant quantities of nitric oxide (NO), pro‐inflammatory factors (TNF‐α, IL‐6, and IL‐12), and chemokines (CXCL1, CXCL2, and CCL2). This activation pathway assumes a pro‐inflammatory role in COPD.^[^ ^7^, ^8^ ^]^ In contrast, the alternative M2 macrophage activation, linked to the Th2 immune response, is induced by Th2 cytokines IL‐4 or IL‐13. This activation pathway upregulates the expression of arginase 1 (Arg1), FiZZ1, and TGF‐β through the activation of JAK‐STAT6 and PI3K/AKT signaling. M2 macrophages play an anti‐inflammatory role while significantly contributing to tissue repair and remodeling in COPD.^[^ ^8^, ^9^ ^]^ Both M1 and M2 macrophages have been identified in COPD and various other lung diseases.^[^ ^1^, ^2^, ^3^, ^4^ ^]^ However, the specific roles of M1 and M2 in COPD are yet to be fully elucidated, and modulating the polarization state of macrophages emerges as a promising therapeutic approach. Notably, research has demonstrated the effectiveness of this approach. For instance, a previous study reported that a nuclear receptor PPAR‐γ agonist markedly alleviated cigarette smoke‐induced airway inflammation in mice by inhibiting M1 macrophage polarization.^[^ ^10^ ^]^ Xu et al. studied that knockdown of RTEL1 relieved COPD development by suppressing M2 macrophage polarization.^[^ ^11^ ^]^ Xu et al. demonstrated that Chinese herb can improve airway remodeling in COPD rats by suppressing M2 macrophage polarization.^[^ ^12^ ^]^ Consequently, exploring novel molecules and signaling pathways involved in macrophage polarization holds significant promise for advancing COPD treatment strategies.

The immunoproteasome, a distinctive proteasomal variant, is constitutively expressed in immune cells, whereas standard proteasomes are active in all somatic cells. In response to proinflammatory cytokines, oxidative stress, and other environmental cues, the constitutive subunits β1, β2, and β5 are replaced by the β1i/LMP2, β2i/MECL‐1, and β5i/LMP7 subunits, forming the immunoproteasome.^[^ ^10^, ^13^ ^]^ The primary function of the immunoproteasome is to generate antigenic peptides, enhancing major histocompatibility class (MHC) I antigen presentation compared to standard proteasomes, and this process facilitates efficient CD8^+^ T cell immune surveillance.^[^ ^14^ ^]^ Additionally, immunoproteasomes modulate the differentiation and activation of lymphocyte subsets, potentially influencing altered transcriptional profiles and cytokine secretion.^[^ ^15^ ^]^ Our previous studies have unveiled the immunoproteasome's regulatory role in modulating AMs polarization.^[^ ^16^ ^]^ Consequently, immunoproteasome activity impacts both adaptive and innate immune cell homeostasis, hinting at the therapeutic potential of manipulating immunoproteasome function in inflammatory diseases. For example, ONX‐0914, a specific immunoproteasome inhibitor, selectively blocks both immunoproteasome subunit sites of β5i/LMP7 and β1i/LMP2.^[^ ^17^ ^]^ Numerous studies have highlighted ONX‐0914′s therapeutic potential in treating inflammatory diseases, including acute viral myocarditis, arthritis, and colitis.^[^ ^17^, ^18^ ^]^ Ilona E Kammerl et al. recently reported that immunoproteasome inhibition reduced pro‐inflammatory cytokine expression in COPD‐derived blood immune cells.^[^ ^19^ ^]^ However, the therapeutic role of ONX‐0914 in COPD‐related airway inflammation remains largely unclear. Therefore, we hypothesize that ONX‐0914 can alleviate COPD airway inflammation by modulating macrophage polarization.

In our investigation, we observed an elevation of both M1 and M2 polarization markers and immunoproteasome subunits LMP2 and LMP7 in macrophages isolated from the LPS + Elastase‐induced experimental emphysema mouse model. We further validated the upregulation of LMP2 and LMP7 in polarized M1 and M2 macrophage induced respectively by LPS/IFNγ and IL‐4 in vitro. Subsequently, we demonstrated that the inhibition of immunoproteasome activity with ONX‐0914 led to a significant alleviation of experimental emphysema inflammation, concomitant with improved lung function, achieved through the inhibition of macrophage polarization. Mechanistically, we elucidated that ONX‐0914 induced the activation of NRF1 and NRF2‐P62 signaling pathways, both of which were crucial for the ONX‐0914‐mediated suppression of M1 polarization. Furthermore, we demonstrated ONX‐0914 inhibited M2 polarization via inhibiting transcription of IRF4. Taken together, the findings from this study strongly advocate for the inhibition of immunoproteasome subunits in macrophages as a promising therapeutic approach for the treatment of COPD.

## 2 Results

### 2.1 Immunoproteasome Elevated in Macrophages from Experimental Emphysema Mice and Polarized macrophages in vitro

**Figure 1:**
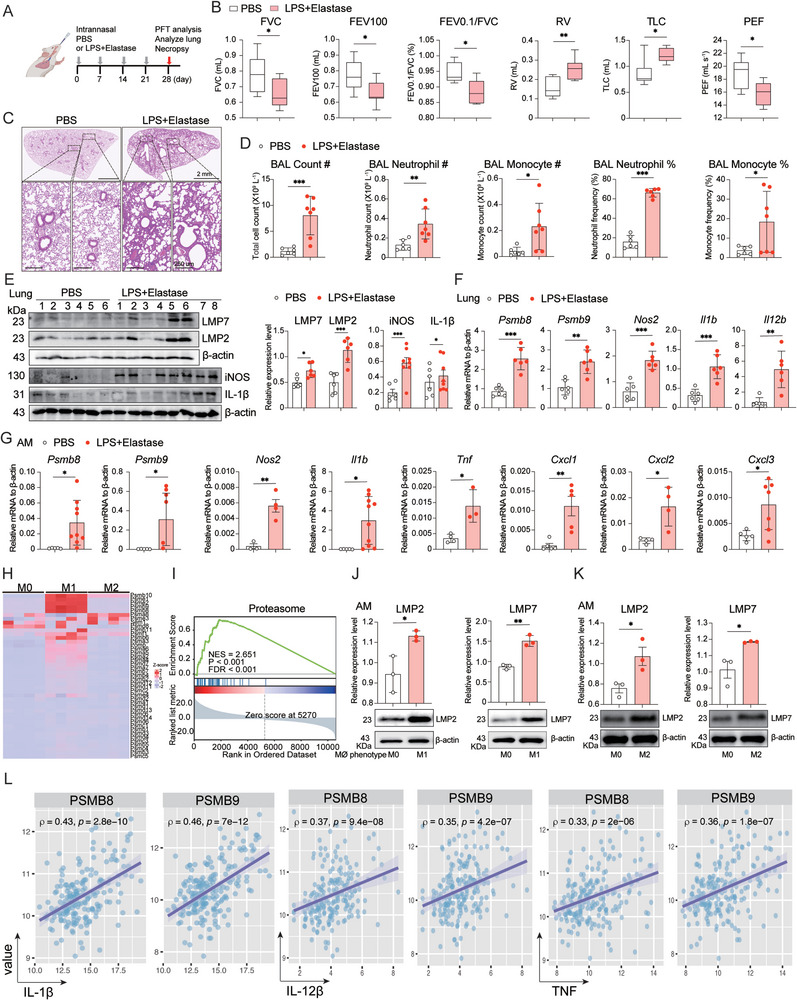
Immunoproteasome Induction in M1 Polarized Macrophages during Experimental Emphysema Development. A) Schematic representation of the COPD mouse model. Briefly, LPS + Elastase was intranasally delivered to C57BL/6JGpt mice weekly for four weeks (n = 7 per group). B) Pulmonary function parameters including Forced Vital Capacity (FVC), Forced Expiratory Volume in 100 milliseconds (FEV100), FEV0.1/FVC ratio (%), Residual Volume (RV), Total Lung Capacity (TLC), and Peak Expiratory Flow (PEF) were measured to evaluate lung function in each group using PFT Pulmonary Maneuvers (n = 7 per group). C) Representative H&E staining images demonstrating enlarged alveolar spaces with abundant inflammatory cell infiltration in the LPS + Elastase group (n = 7 per group). D) Total cell counts in bronchoalveolar lavage (BAL) fluid, including neutrophils and monocytes, were detected in each group using a Sysmex blood analyzer (n = 7 per group). E) Expression levels of immunoproteasome subunits LMP2, LMP7, and M1 marker proteins iNOS, IL‐1β were analyzed in lung tissue of each group by Western blotting analysis. Mean values ± SEM from at least 6 independent samples is presented. F) RT‐qPCR analysis examined the mRNA expression of immunoproteasome subunits *Psmb8*, *Psmb9*, and M1 marker genes *Nos2*, *Il1b*, and *Il12b* in lung tissue of each group, respectively, and n = 6 for each group. G) RT‐qPCR analysis detected Psmb8, Psmb9, and M1 marker genes, including *Nos2*, *Il1b*, *Tnf*, *Cxcl1*, *Cxcl2*, and *Cxcl3*, in alveolar macrophages (AMs) of each group. Data was collected from 6 independent samples. H) Bulk RNA‐seq transcriptomes showing the differences in immunoproteasome subunit‐related genes between M0, M1, and M2 phenotype of peritoneal macrophages (PMs) isolated from 8‐week‐old C57BL/6JGpt mice (n = 4 independent samples in each group). I) Hallmark gene sets with significant changes in Gene Set Enrichment Analysis (GSEA) between M1 and M0 phenotype of PMs. False Discovery Rate (FDR) q < 0.05 was considered significant (n = 4 independent samples for each group). J and K) Western blotting analysis showing expression levels of immunoproteasome subunits LMP2 and LMP7 in M0 and M1 (J), M0 and M2 (K) phenotype of AMs, respectively. Data were expressed as means ± SEM from 3 independent experiments. L) The relationship between M1 marker genes IL‐1β, IL‐12β, TNF, and immunoproteasome‐related genes (Psmb8, Psmb9) was reanalyzed using a clinical cohort of sputum transcriptomes from 99 patients with COPD and 36 healthy individuals in China. *p < 0.05, **p < 0.01, ***p < 0.001.

**Figure 2:**
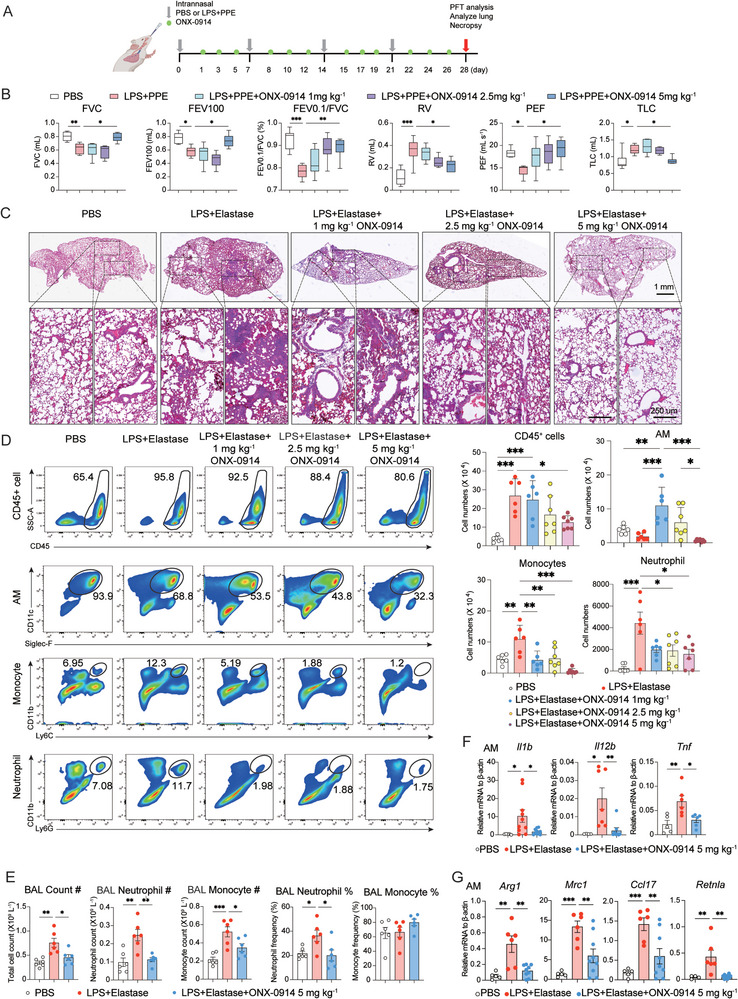
Immunoproteasome Inhibition as a Therapeutic Approach for Emphysema. A) Experimental setup for the COPD experiment and n ≥ 6 for each group. ONX‐0914 intranasal treatment was initiated one day post‐LPS + Elastase administration, administered three times weekly for four weeks to induce COPD. Gradient dosages of ONX‐0914 (1 mg kg^−1^, 2.5 mg kg^−1^, 5 mg kg^−1^) were administered one day post‐LPS + Elastase, three times weekly for 4 weeks. B) Pulmonary function indexes, including FVC, FEV100, FEV0.1/FVC%, RV, PEF, and TLC, were tested in each group using PFT Pulmonary Maneuvers. C) H&E staining observed the alveolar changes in lung tissue in each group. D) Cellular infiltration in BAL fluid was detected in each group by flow cytometry analysis. E) Total BAL cell counts, including neutrophils and monocytes, were measured using a Sysmex blood analyzer in the control, LPS + Elastase, and 5 mg kg^−1^ ONX‐0914 treatment groups. F) RT‐qPCR analysis detected M1 marker genes *Il1b, Il2b, and Tnf* in alveolar macrophages (AMs) of each group. Data was collected from 6 independent samples. G) M2 marker genes, such as *Arg1*, *Mrc1*, *Ccl17*, and *Retnla*, were detected with RT‐qPCR analysis in AMs of each group. Data was collected from 6 independent samples. *p < 0.05, **p < 0.01, ***p < 0.001.

To investigate the potential therapeutic target of immunoproteasome for COPD, we established an experimental murine model for emphysema through intranasal administration of LPS + Elastase (**Figure** **1**A). Pulmonary function tests revealed a significant decrease in forced expiratory volume at 100 milliseconds (FEV100), forced vital capacity (FVC), FEV0.1/FVC (%) and residual volume (RV), but increased total lung capacity (TLC) and peak expiratory flow (PEF) in the LPS + Elastase group compared with the PBS group (Figure 1B). Histological assessments indicated enlarged alveolar spaces accompanied by abundant inflammatory cell infiltration in the LPS + Elastase group (Figure 1C; Figure S1G, Supporting Information). Moreover, LPS and Elastase induced the infiltration of neutrophils and monocytes in the bronchoalveolar lavage (BAL) fluid, consistent with the histological findings (Figure 1D). Through Western blotting, we observed a significant upregulation of immunoproteasome subunits LMP2 and LMP7 at the protein level, along with increased expression of M1 markers iNOS and IL‐1β in the LPS + Elastase group (Figure 1E). These results were further confirmed at the mRNA level using RT‐qPCR in lung tissue (Figure 1F). Meanwhile, M2 marker genes, including *Arg1*, *Mrc1*, *Ccl17*, and *Retnla*, were also increased in lung tissue of LPS + Elastase group (Figure S2D, Supporting Information). Furthermore, we analyzed the publicly available scRNA‐seq data of human lung from Human Protein Atlas (www.proteinatlas.org) and found that the expression of PSMB9 and PSMB8 in macrophages was significantly higher compared to it in alveolar epithelial cells (Figure S1A,B, Supporting Information). RT‐qPCR analysis of different macrophages (AM, PM, and RAW264.7 cell line) and alveolar epithelial cells (MEL12 cell line) confirmed the same conclusion (Figure S1C, Supporting Information). These results indicated that the immunoproteasome subunits are predominantly expressed in macrophages, but not epithelial cells, and therefore we focused on the role of immunoproteasome in macrophages but not epithelial cells (Figure S1D, Supporting Information). Next, isolated macrophages from BAL fluid in the LPS + Elastase group exhibited induced expression of *Psmb8/Psmb9*, M1 (*Nos2*, *Il1b*, *Tnf*, *Cxcl1*, *Cxcl2*, and *Cxcl3*) and M2 marker genes (*Arg1*, *Mrc1*, *Ccl17*, and *Retnla*) compared to the control group (Figures 1G and 2G), indicating a correlation between immunoproteasome and macrophage activation during emphysema development. Moreover, we found other proteasome subunits (*Psmb5*, *Psmb6*, *Psmb7* and *Psmb10*) were not as highly expressed as *Psmb8* and *Psmb9* in alveolar macrophages (AMs) and lung tissue of LPS + Elastase models (Figure S1E,F, Supporting Information). To explore the relationship between immunoproteasome and macrophage polarization in vitro, we conducted bulk RNA‐seq analysis on peritoneal macrophages (PMs) undergoing M1 and M2 polarization. Heat map analysis revealed higher expression of immunoproteasome subunit‐related genes, including *Psmb8, Psmb9, Psmb10, Psme1, Psme2*, *Psme2b*, *and Psme6*, in M1 compared to M0 macrophages. Some of them were also enriched in M2, but the mRNA of *Psmb8* and *Psmb9* were not highly expressed in M2 macrophages (Figure 1H). Gene set enrichment analysis (GSEA) of hallmark gene sets further confirmed the increased expression of proteasome‐related genes in M1‐polarized macrophages (Figure 1I). Subsequently, we validated the elevated protein expression of LMP2 and LMP7 both in M1 and M2 polarized AMs through Western blotting (Figure 1J and K). Although LMP2 and LMP7 were not consistent at the level of transcription and protein translation, the highly expressed protein levels could still indicate that the immunoproteasome subunit was closely related to M2 polarization. Taken together, these results reconfirmed the positive correlation between the immunoproteasome and macrophage polarization.

Furthermore, to assess the clinical relevance of our findings, we examined the connection between immunoproteasome subunits and macrophage polarization markers by reanalyzing a clinical cohort of sputum transcriptomes from 99 patients with COPD and 36 healthy individuals in China. Our analysis unveiled a positive correlation between the transcriptional levels of macrophage polarization markers (IL‐1β, IL‐12β, TNF, CXCL1, and CXCL2) and LMP2 and LMP7 (EGAS00001006398) (Figure 1L; Figure S1H, Supporting Information).^[^ ^20^ ^]^ These findings collectively suggest that the expression of immunoproteasome is heightened concomitantly with macrophage polarization during the development of COPD.

### 2.2 Immunoproteasome Inhibition as a Therapeutic Approach for Emphysema

To assess the therapeutic potential of immunoproteasome in polarized macrophages as a target for emphysema, we intratracheally administered ONX‐0914, a selective inhibitor of immunoproteasome subunits LMP7 and LMP2, into the lungs of emphysema‐induced mice three times a week for four consecutive weeks, beginning one day after the initiation of LPS + Elastase treatment, at various dosages (1, 2.5, and 5 mg kg^−1^) (**Figure** **2**A). Using pulmonary function tests, we initially observed that ONX‐0914 at a dosage of 5 mg kg^−1^ significantly improved FEV100, FVC, FEV0.1/FVC (%) and PEF, but suppressed RV and TLC in mice with LPS + Elastase‐induced emphysema (Figure 2B). Subsequently, H&E staining demonstrated that ONX‐0914 markedly alleviated lung inflammation in emphysema mice, although it did not affect the expanded alveolar spaces (Figure 2C; Figure S2A, Supporting Information). To investigate the effects of ONX‐0914 on immune responses relevant to emphysema, we employed spectral flow cytometry and a hematology analyzer to determine the number and percentage of inflammatory cells in BAL fluid. We found that ONX‐0914 (5 mg kg^−1^) treatment significantly inhibited the infiltration of neutrophils and monocytes in BAL fluid (Figure 2D and E).

To explore whether the therapeutic efficacy of ONX‐0914 was contingent on its inhibitory effect on macrophage polarization, we conducted Western blotting and RT‐qPCR analysis of M1 markers at the protein and mRNA levels in lung tissue. Remarkably, treatment with 5 mg kg^−1^ of ONX‐0914 significantly suppressed the protein expression of iNOS and the mRNA levels of *Nos2*, *Il1b*, and *Il12b* (Figure S2B,C, Supporting Information). To further validate the inhibitory role of immunoproteasome in M1 macrophage polarization, we isolated BAL fluid macrophages and examined the gene expression of M1 markers. Our findings demonstrated a clear inhibition of *Il1b*, *Il12b*, and *Tnf* expression in macrophages following ONX‐0914 treatment (Figure 2F). Intriguingly, we also found ONX‐0914 could effectively suppress M2 polarization in AMs and lung tissue of COPD model (Figure 2G; Figure S2D, Supporting Information). In summary, these results strongly indicated that immunoproteasome inhibition holds significant therapeutic potential for emphysema treatment by modulating macrophage polarization.

### 2.3 Immunoproteasome Inhibition Represses Macrophages Polarization in vitro

**Figure 3:**
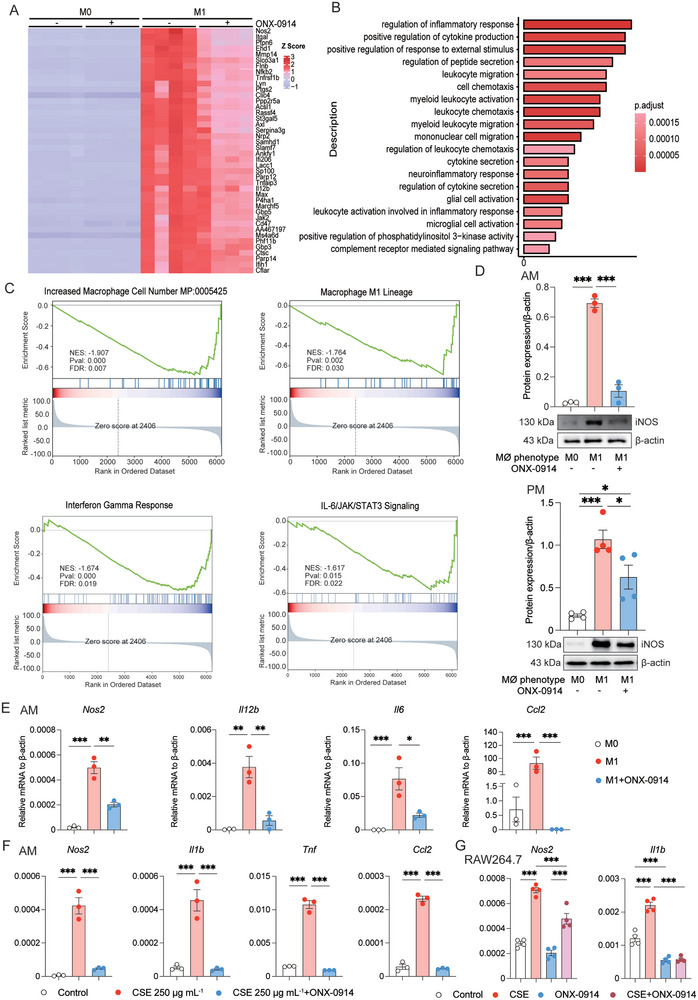
Immunoproteasome Inhibition Suppresses M1 Macrophage Polarization In Vitro. A) Heat maps showing M1 marker genes in PMs in ONX‐0914 and non‐ONX‐0914 groups, including M0, M0+ONX‐0914, M1, M1+ONX‐0914 (n = 4 independent samples in each group). B) GO enrichment analysis of genes downregulated by ONX‐0914 treatment in M1. The y‐axis represents the GO terms, and the x‐axis indicates the gene count. C) GSEA for Differentially Expressed Genes (DEGs) of M1 after ONX‐0914 treatment. The y‐axis represents the enrichment score, and the x‐axis is the ranked list of genes from highest to lowest based on statistical significance after ONX‐0914 treatment. The results show that the increased macrophage cell number, macrophage M1 lineage, IL‐6/JAK/STAT3, and IFN‐γ pathways were significantly repressed by ONX‐0914. D) After pretreatment with 0.2 µM ONX‐0914 for 6 h, the M1 marker protein iNOS was analyzed by Western blotting in M1 polarization of AMs and PMs (n = 3 in each group). E) After pretreatment with 0.2 µM ONX‐0914 for 6 h, the M1 marker genes *Nos2, Il12b, Il6, and Ccl2* were examined by qPCR in AMs (n = 3 in each group). F) Pre‐treated with 0.2 µM ONX‐0914 for 6 h, AMs were challenged with 250 µg mL^−1^ dosage of CSE for 24 h, and the M1 marker genes *Nos2, Il1b, Tnf, and Ccl2* were examined by RT‐qPCR (n = 3 in each group). G) Pre‐treated with 0.2 µM ONX‐0914 for 6 h, RAW264.7 cell line was challenged with 40 µg mL^−1^ CSE for 24 h, and the M1 marker genes *Nos2, Il1b* were examined by RT‐qPCR (n = 4 in each group). *p < 0.05, **p < 0.01, ***p < 0.001.

**Figure 4:**
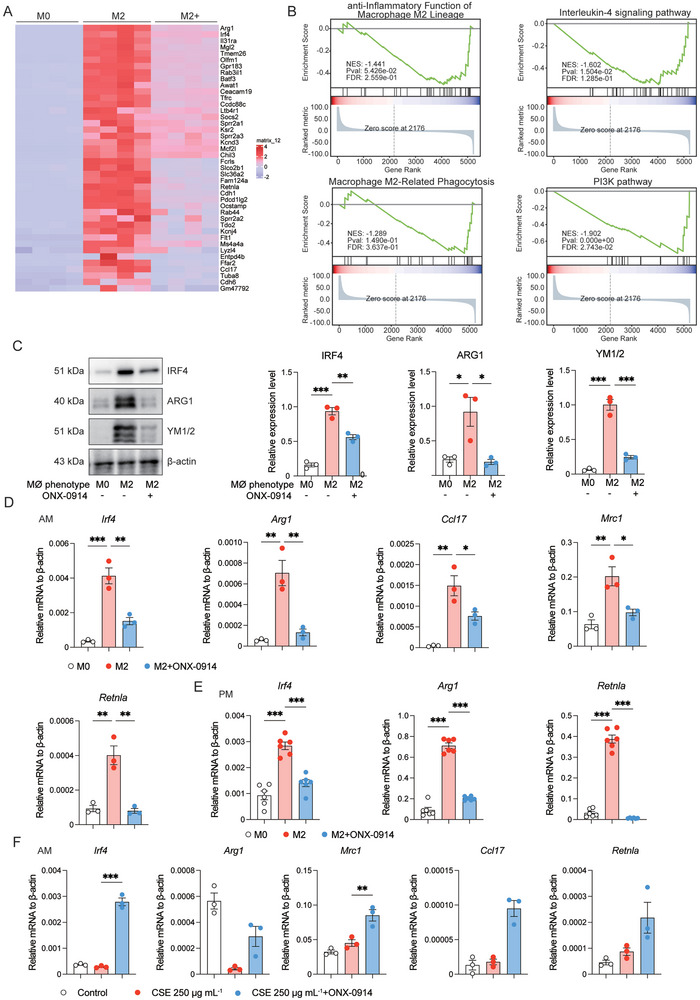
Immunoproteasome Inhibition Suppresses M2 Macrophage Polarization In Vitro. A) Heat map shows M2 marker genes in PMs in ONX‐0914 and non‐ONX‐0914 groups, including M0, M0+ONX‐0914, M2, M2+ONX‐0914 (n = 4 independent samples in each group). B) GSEA) for DEGs of M2 after ONX‐0914 treatment. The y‐axis represents the enrichment score, and the x‐axis is the ranked list of genes from highest to lowest based on statistical significance after ONX‐0914 treatment. The results show that the anti‐inflammatory function of macrophage M2 lineage, interleukin‐4 signaling pathway, macrophage M2‐related phagocytosis, PI3K pathway were significantly repressed by ONX‐0914. C) After pre‐treated with 0.2 µM ONX‐0914 for 6 h, the M2 marker protein IRF4, ARG1, YM1/2 was analyzed by Western blotting in M2 polarization of PMs (n = 3 in each group). D) After pre‐treated with 0.2 µM ONX‐0914 for 6 h, the M2 marker genes *Irf4*, *Arg1*, *Ccl17*, *Mrc1* and *Retnla* were examined by RT‐qPCR in AMs (n = 3 in each group). E) After pretreatment with 0.2 µM ONX‐0914 for 6 h, the M2 marker genes *Irf4*, *Arg1*, and *Retnla* were examined by RT‐qPCR in PMs (n = 3 in each group). F) Pre‐treated with 0.2 µM ONX‐0914 for 6 h, AMs were challenged with 250 µg mL^−1^ dosage of cigarette smoke extract (CSE) for 24 h, and the M2 marker genes *Irf4*, *Arg1*, *Mrc1*, *Ccl17* and *Retnla* were examined by RT‐qPCR (n = 3 in each group). *p < 0.05, **p < 0.01, ***p < 0.001.

As demonstrated above, our findings highlighted the inhibitory effect of ONX‐0914 on macrophage polarization during emphysema development in vivo. To delve deeper into the impact of immunoproteasome inhibition on M1 and M2 macrophage polarization in vitro, we isolated murine primary PMs and induced to M1 or M2 phenotype using IFN‐γ (20 ng mL^−1^)/LPS (1000 ng mL^−1^) or IL‐4 (10 ng mL^−1^), respectively. Subsequently, we performed bulk RNA‐seq to comprehensively analyze changes in the transcriptome in response to ONX‐0914 treatment. The heat map analysis revealed that ONX‐0914 treatment significantly blocked the expression of several IFN‐γ/LPS‐induced M1 genes in macrophages. Notable genes were affected included *Nos2*, *Itgal*, *Ptpn6*, and *IL12b*, among others (**Figure** **3**A). Similarly, the downregulation of marker genes, such as *Arg1*, *Irf4*, *Retnla* and *Ccl17*, in M2 after ONX‐0914 administration was visualized by heat maps (**Figure** **4**A). Gene ontology (GO) enrichment analysis unveiled that ONX‐0914 suppressed biological pathways mainly associated with immune activation, including the regulation of inflammation responses, positive regulation of cytokine production, and responses to external stimuli, among others (Figure 3B). Gene set enrichment analysis (GSEA) of hallmark gene sets further confirmed the inhibition of macrophage cell number, macrophage M1 lineage, and M1‐related inflammatory pathways (Interferon Gamma Response and IL‐6/JAK/STAT3 signaling) by ONX‐0914 (Figure 3C). GSEA analysis also showed that the anti‐inflammatory function of macrophage M2 lineage, interleukin‐4 signaling pathway, macrophage M2‐related phagocytosis, PI3K pathway were repressed in M2 by ONX‐0914 (Figure 4B). Moreover, ONX‐0914 treatment significantly inhibited iNOS protein expression both in M1 AMs and PMs (Figure 3D). Meanwhile, the mRNA levels of *Nos2*, *Il1b*, and *Il12b* were decreased by ONX‐0914 in M1 AMs (Figure 3E). Western blotting revealed a noticeable gel shift of LMP2 and LMP7 in ONX‐0914‐treated AMs, indicating the co‐inhibition of LMP2 and LMP7 by ONX‐0914 (Figure S2E, Supporting Information). Correspondingly, ONX‐0914 also inhibited the expression of M2 markers at both protein (IRF4, ARG1, YM1/2) and mRNA (*Arg1*, *Irf4*, *Ccl17*, *Mrc1* and *Retnla*) levels in IL‐4 induced M2 phenotype macrophage (Figure 4C–E). Additionally, we challenged AMs with cigarette smoke extract (CSE) at a dosage of 250 µg mL^−1^ to simulate COPD inflammatory responses in vitro, and examined the effect of ONX‐0914 on this process. We discovered that CSE significantly increased M1 markers, while M2 markers did not show a notable elevation. Moreover, pretreatment with ONX‐0914 (0.2 µM) for 6 h could markedly suppress the elevated M1 markers (*Nos2*, *Il1b*, *Tnf*, and *Ccl2*) (Figure 3F), but had an opposite effect on M2 markers (*Arg1*, *Irf4*, *Ccl17*, *Mrc1* and *Retnla*) (Figure 4F). Consistently, ONX‐0914 significantly suppressed the CSE‐induced upregulation of *Nos2* and *Il1b* in RAW264.7 cells (Figure 3G). These results provided substantial evidence that ONX‐0914 can inhibit the polarization of both M1 and M2 macrophages in vitro. However, in the CSE model, its primary effect is to mitigate inflammation by suppressing M1 polarization not M2 polarization.

### 2.4 Autophagy and Endoplasmic Reticulum (ER) Stress were not Attribute to the ONX‐0914 Mediated Suppression of Macrophage Polarization

To delve into the underlying mechanisms by which ONX‐0914 suppresses macrophage polarization, we analyzed the RNA‐seq datasets from PMs and identified enrichment in macro‐autophagy activation genes upon ONX‐0914 stimulation (Figure S3A, Supporting Information). Our observations have revealed that ONX‐0914 induced the upregulation of the key autophagy activation marker LC3 II, peaking at 24 h in various macrophages, including PMs, AMs, and RAW264.7 cells, as demonstrated by Western blotting (Figure S3B, Supporting Information). We further investigated autophagy activation using the Tandem fluorescent‐tagged LC3 assay, confirming increased LC3B‐positive puncta accumulation, indicating autophagy activation by ONX‐0914 in AMs (Figure S3C, Supporting Information). The same results were found in PMs by using LC3B antibody based immunofluorescent assay (Figure S3D, Supporting Information). Moreover, similar activation of autophagy by ONX‐0914 was confirmed both in M1 and M2 macrophages (Figure S3E,F, Supporting Information). It has been previously reported that activation of autophagy can inhibit the macrophage activation.^[^ ^21^ ^]^ Given that autophagy can negatively regulate macrophage M1 and M2 polarization, we hypothesized that ONX‐0914′s inhibition of macrophage polarization might rely on autophagy activation. To explore this, we investigated if chloroquine (CQ), a conventional end‐stage autophagy inhibitor, could rescue the expression of M1 and M2 markers in polarized macrophages pretreated with ONX‐0914. Surprisingly, contrary to our expectations, treatment with CQ enhanced ONX‐0914′s inhibitory effect on both M1 and M2 polarization in AMs rather than rescue it. (Figure S3G,H Supporting Information). Additionally, a CCK8 assay showed that ONX‐0914 significantly decreased macrophage viability, and this effect was further intensified by co‐treatment with CQ in M1 (Figure S3I, Supporting Information). At the same time, RT‐qPCR analysis had also reaffirmed that CQ exacerbated the inhibitory effect of ONX‐0914 on M2 polarization in AMs (Figure S3JA, Supporting Information). The aforementioned results indicated that ONX‐0194 can activate autophagy, but the inhibition of M1 and M2 polarization is not dependent on autophagy.

In addition to the induction of autophagy, our bulk RNA‐seq analysis revealed an activation of endoplasmic reticulum (ER) stress upon ONX‐0914 stimulation in macrophages by heatmap and GSEA (Figure S4A,B, Supporting Information). Proteasome inhibition has been well‐known to induce the accumulation of misfolded proteins and ER stress.^[^ ^22^, ^23^ ^]^ To confirm this, we used Western blotting to analyze key ER stress markers and found that protein level of ubiquitin (Ub) was increased in a dosage‐dependent manner, and the high expression of Bip and GADD153 (CHOP) had a time‐dependent manner in RAW264.7 cell line (Figure S4C,D, Supporting Information). Besides, 0.2 µM ONX‐0914 stimulated the upregulation of phosphorylation level of eIF2α and JNK1 in a time‐dependent manner in AMs (Figure S4E, Supporting Information). Furthermore, immunofluorescence assays revealed that ONX‐0914 treatment for 24 h significantly induced aggregation of GFP‐KDEL in ER of AMs by using a piggyBac transposon‐based expression system, implying ER stress was activated in comparison with control group (Figure S4F, Supporting Information). Previous studies have suggested a regulatory role of ER stress in macrophage polarization.^[^ ^24^ ^]^ To investigate if ONX‐0914 inhibits M1 polarization through activating ER stress, we tested the effect of 100 nM 4‐Phenylbutyric acid (4‐PBA), an ER stress inhibitor, on ONX‐0914‐treated M1 macrophages. Surprisingly, 4‐PBA did not rescue the down‐regulation of iNOS in ONX‐0914‐treated M1 macrophages (Figure S4G, Supporting Information). Taken together, these results demonstrate that ONX‐0914 mediated suppression of M1 polarization was not dependent on activation of autophagy or ER stress.

### 2.5 ONX‐0914 Suppresses M1 Polarization via NRF1 and NRF2‐P62 Signaling Pathway

**Figure 5:**
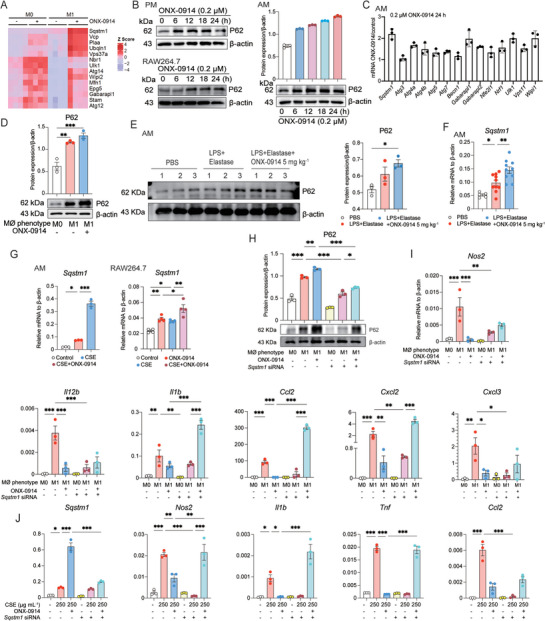
P62 Plays a Key Role in ONX‐0914‐Mediated Suppression of M1 Polarization. A) Heatmaps displaying autophagy‐related genes, especially *Sqstm1*, in various groups of PMs, including M0, M0+ONX‐0914, M1, and M1+ONX‐0914 (n = 4 independent samples in each group). B) Protein expression levels of P62 were analyzed in PM, AM and RAW264.7 cell line after 0.2 µM ONX‐0914 treatment at different time points by Western blotting analysis (n = 3). C) Autophagy‐related genes were detected after ONX‐0914 treatment in AMs. Mean values ± SEM are acquired from 3 independent experiments. D) After pretreatment with 0.2 µM ONX‐0914 for 6 h, the expression level of P62 was analyzed by Western blotting in M1‐polarized AMs. Statistical analysis were obtained from 3 independent samples in each group. E) The expression of P62 was analyzed in lung tissue after 5 mg kg^−1^ ONX‐0914 treatment in COPD by Western blotting (n = 3). F) RT‐qPCR analysis detected *Sqstm1* expression in AMs after 5 mg kg^−1^ ONX‐0914 treatment in COPD mice. Mean values ± SEM are from at least 5 independent experiments. G) RAW264.7 cells and AMs were pretreated with 0.2 µM ONX‐0914 for 6 h, followed by stimulation with 40 µg mL^−1^ and 250 µg mL^−1^ doses of cigarette smoke extract (CSE) for 24 h, respectively. The gene *Sqstm1* was examined by RT‐qPCR (n = 4). H) After 0.2 µM ONX‐0914 treatment in M1‐polarized AMs and silencing of P62 expression with siRNA, the expression of P62 was analyzed by Western blotting (n = 3). I) In M1‐polarized AMs, after silencing the expression of P62 with siRNA and treating with 0.2 µM ONX‐0914, the expression of M1 marker genes (*Nos2, Il12b, Il1b, Ccl2, Cxcl2, Cxcl3*) was detected by RT‐qPCR (n = 3). J) After knockdown the expression of P62 with siRNA and pretreating with 0.2 µM ONX‐0914, AMs were stimulated with CSE (250 µg mL^−1^). The expression of *Sqstm1* and M1 marker genes (*Nos2, Il1b, Tnf, Ccl2*) was detected by RT‐qPCR (n = 3). *p < 0.05, **p < 0.01, ***p < 0.001.

To further explore out underlying molecular mechanism by which ONX‐0914 suppresses macrophage polarization, we analyzed the RNA‐seq dataset and discovered that ONX‐0914 markedly induced the expression of P62 and other autophagy‐related genes in PMs (**Figure** **5**A). P62 is a multifunctional adaptor protein implicated in various cellular processes, including selective autophagy, proteasome function, cell survival, oxidative stress response, and inflammation.^[^ ^25^, ^26^ ^]^ Previous studies have shown that P62 can serve as a negative regulator of M1 macrophage polarization.^[^ ^27^, ^28^ ^]^ Therefore, we speculated that P62 might act as a downstream molecule of ONX‐0914 stimulation, contributing to the regulation of M1 polarization. A time‐course analysis was conducted to investigate the expression dynamics of P62. We found that ONX‐0914 induced a time‐dependent upregulation of P62 protein levels in PMs, AMs, and RAW264.7 cells from 6 to 24 h (Figure 5B). RT‐qPCR analysis further confirmed ONX‐0914‐induced upregulation of P62 and other autophagy‐related genes in AMs (Figure 5C). Moreover, we observed that ONX‐0914 enhanced M1 polarization‐induced P62 upregulation at the protein level in AMs (Figure 5D). Additionally, these findings were verified with a mouse model, where LPS + Elastase treatment‐induced upregulation of P62 at both the protein and mRNA levels was further enhanced by ONX‐0914 in AMs of BAL fluid (Figure 5E and F; Figure S2F,G, Supporting Information). RT‐qPCR results also demonstrated that ONX‐0914 enhanced CSE‐stimulated P62 induction in AMs and RAW264.7 cells (Figure 5G).

To investigate the specific role of P62 in the regulation of M1 polarization under ONX‐0914 stimulation, we conducted functional rescue experiments using RNA interference technology. Western blotting assays confirmed the efficiency of siRNA‐mediated P62 knockdown (Figure 5H; Figure S2H, Supporting Information). Subsequently, we found that P62 siRNA partially reversed ONX‐0914‐mediated suppression of M1 marker gene expression (*Nos2*, *Il1b*, *Il12b*, *Ccl2*, *Cxcl2* and *Cxcl3*) in AMs (Figure 5I). These results were consistent with the observation that P62 siRNA also partially rescued the ONX‐0914‐mediated suppression of M1 markers in CSE‐treated AMs (Figure 5J).

**Figure 6:**
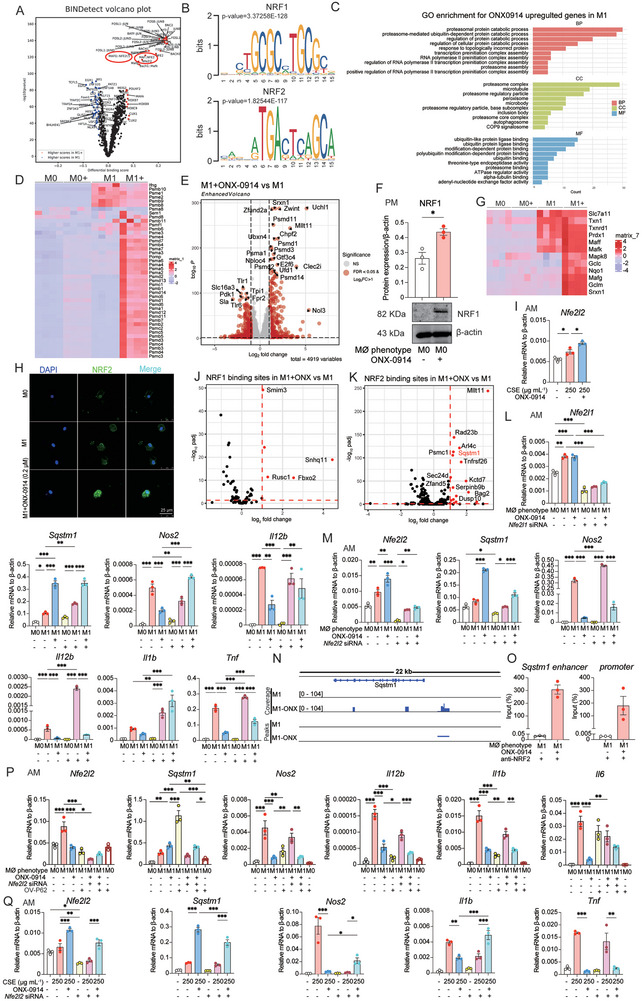
ONX‐0914 Suppresses M1 Polarization via NRF1 and NRF2‐P62 Signaling Pathway. A) Differential binding of transcription factors between M1+ONX‐0914 and M1 predicted by TOBIAS. This plot indicates a significant increase in NRF1 (MAFG::Nfe2l1) and NRF2 (MAF::Nfe2l2) binding score following ONX‐0914 treatment. B) Mouse genome NRF1 and NRF2 motifs obtained from JASPAR 2022 database. C) Gene Ontology enrichment analysis of genes upregulated by ONX‐0914 treatment in M1 phenotype of PMs. The y‐axis represents the GO terms, and the x‐axis indicates the gene count. D) Heatmaps displaying proteasome‐related genes, the NRF1 downstream regulated genes, in M0, M0+ONX‐0914, M1, and M1+ONX‐0914 group (n = 4). E) Volcano plot showing DEGs between ONX‐0914‐treated and untreated M1 AMs. Genes meeting dual thresholds of FDR <0.05 and log_2_ (fold change) >1 are highlighted in red, indicating significant upregulation or downregulation after ONX‐0914 treatment. F) Protein expression level of NRF1 analyzed by Western blotting in ONX‐0914‐treated M1 AMs. Data was obtained from 3 independent samples in each group. G) Heatmaps displaying anti‐oxidative stress‐related genes, the NRF2 downstream regulated genes, in M0, M0+ONX‐0914, M1, and M1+ONX‐0914 group of PMs (n = 4). H) Representative IF images of NRF2 after applying 0.2 µM ONX‐0914 to AMs for 24 h. I) After AMs were pretreated with 0.2 µM ONX‐0914 for 6 h, and then stimulated with 250 µg mL^−1^ dosage of CSE for 24 h, the gene *Nfe2l2* was examined by RT‐qPCR (n = 3). J and K) Transcriptional changes in genes associated with NRF1‐increased binding sites (J) and NRF2‐increased binding sites (K) after ONX‐0914 treatment in M1 of AMs. The plot demonstrates that the regulatory activity of upregulated genes significantly surpasses that of downregulated genes (n = 3). L) After silencing the expression of NRF1 with siRNA and treating M1‐polarized AMs with 0.2 µM ONX‐0914, the expression of *Nfe2l1, Sqstm1*, and M1 marker genes (*Nos2, Il12b*) were detected by RT‐qPCR (n = 3). M) After silencing the expression of NRF2 with siRNA and treating M1‐polarized AMs with 0.2 µM ONX‐0914, the expression of *Nfe2l2, Sqstm1*, and M1 marker genes (*Nos2, Il12b, Il1b, Tnf*) were detected by RT‐qPCR (n = 3). N) Integrative Genomics Viewer (IGV) plot illustrates the binding landscape of the NRF2 transcription factor to *Sqstm1* in M0, M0+ONX‐0914, M1 and M1+ONX‐0914. The upper 4 tracks display the signal intensity of NRF2 binding to *Sqstm1*, and the lower 4 tracks display the specific locations where NRF2 binding peaks were identified, which suggest the potential regulatory roles in Sqstm1 gene expression after ONX‐0914 treatment in M1 macrophages. O) CUT&Tag‐qPCR shows that the binding affinity of NRF2 to *Sqstm1* enhancer and promoter is significantly increased under ONX‐0914 treatment in M1 macrophages. P) RT‐qPCR analysis detected *Nfe2l2*, *Sqstm1* and M1 marker genes *Nos2*, *Il12b*, *Il1b*, and *Il6* in each group (n = 3). After silencing the expression of NRF2 with siRNA and overexpression of P62 with p3xFLAG‐P62 var2 plasmid, 0.2 µM ONX‐0914 was used to treat M1‐polarized AMs, and the expression of *Nfe2l2*, *Sqstm1*, and M1 marker genes (*Nos2*, *Il12b*, *Il1b* and *Il6*) were detected by RT‐qPCR (n = 3). Q) After silencing the expression of NRF2 with siRNA and using 0.2 µM ONX‐0914 pretreated CSE (250 µg mL^−1^) stimulation of AMs, *Sqstm1* and M1 marker genes (*Nos2, Il12b, Il1b, Tnf*) were detected by RT‐qPCR (n = 3). *p < 0.05, **p < 0.01, ***p < 0.001.

A crucial aspect of gene regulation involves transcription factors (TFs) binding to promoter regions, initiating the expression of target genes. To elucidate the TFs acting as upstream regulators of P62, we employed ATAC‐seq and discovered the activation of both nuclear factor erythroid 2‐related factor 1 and 2 (NFE2L1 and NFE2L2, or NRF1 and NRF2) in ONX‐0914‐stimulated macrophages through motif enrichment analysis of differential peaks (**Figure** **6**A and B). Nrf1 and Nrf2 are essential in various cellular functions, including oxidative stress response, autophagy, proteasome homeostasis, and the negative regulation of M1 polarization.^[^ ^29^, ^30^ ^]^ To validate the activation of Nrf1 by ONX‐0914, we analyzed Nrf1 downstream target genes and pathways using RNA‐seq. Heat map, GO, and volcano plot analysis revealed the induction of several constitutive proteasome subunit genes, known Nrf1 targets, indicating Nrf1 activation by ONX‐0914 (Figure 6C‐E). Western blotting further confirmed NRF1 activation upon ONX‐0914 stimulation (Figure 6F). Similarly, RNA‐seq analysis further demonstrated the upregulation of a group of Nrf2 target genes in macrophages treated with ONX‐0914, indicating Nrf2 activation (Figure 6G). This activation was further supported by increased nuclear localization of NRF2 following ONX‐0194 treatment, as shown by NRF2 antibody‐based fluorescent staining (Figure 6H). RT‐qPCR results also demonstrated that ONX‐0914 enhanced CSE‐stimulated Nrf2 expression in AMs (Figure 6I). Additionally, using Transcription Factor Occupancy By Investigation of ATAC‐seq Signal (TOBIAS) analysis, we predicted the Nrf1 and Nrf2 binding sites in ONX‐0194‐treated M1 macrophages. Interestingly, Nrf2, but not Nrf1, was identified as binding to the P62 gene regulatory site (Figure 6J and K).

To confirm our findings, we employed siRNA targeting Nrf1 and Nrf2. Intriguingly, Nrf2 siRNA significantly inhibited ONX‐0914‐induced expression of P62, while Nrf1 siRNA did not have a significant effect (Figure 6L and M). Surprisingly, silencing both Nrf1 and Nrf2 partially restored ONX‐0914‐suppressed M1 marker gene expression (Figure 6L and M). To further investigate if NRF2 server as transcription factor to initiate the *Sqstm1* transcription by directly binding to gene upstream regulatory site, we applied CUT&Tag, a new technique advanced ChIP‐seq to for mapping specific transcription factor (TF)‐DNA interactions. The CUT&Tag data showed that the intensity of NRF2 binding to the upstream gene regulatory site of *Sqstm1* was significantly increased in M1 + ONX‐0914, which was also corroborated by peak predictions made by SEACR (Figure 6N). Further CUT&Tag‐qPCR also confirmed that the binding affinity of NRF2 to both the enhancer and promoter regions of the *Sqstm1* gene was markedly elevated in M1 + ONX‐0914 (Figure 6O). Moreover, in order to elucidate NRF2 regulated P62 was required for ONX‐0914 mediated M1 inhibition, we overexpressed P62 by transfecting a p62 vector to recovery NRF2 siRNA mediated down‐regulation of P62 under the condition of ONX‐0914 treatment. Compared to silencing NRF2 alone, we observed that NRF2 siRNA mediated recovery of M1 marker genes (*Nos2*, *Il12b*, *Il1b* and *Il6*) were repressed again by overexpressing P62 under the condition of ONX‐0914 treatment of M1 polarized macrophage (Figure 6P). This results further validated that the inhibitory effect of ONX‐0914 on M1 polarization was through NRF2‐P62 axis. Furthermore, Nrf2 siRNA inhibited ONX‐0914‐induced upregulation of P62 in CSE‐treated AMs, thereby partially restoring the ONX‐0914‐induced suppression of CSE‐induced gene expression of *Nos2*, *Il1b*, and *Tnf* (Figure 6Q).

In summary, our findings reveal the intricate involvement of both NRF1 and NRF2‐P62 signaling pathways in the regulation of M1 polarization upon immunoproteasome inhibition.

### 2.6 ONX‐0914 Suppresses M2 Polarization via Inhibiting Transcription of IRF4

**Figure 7:**
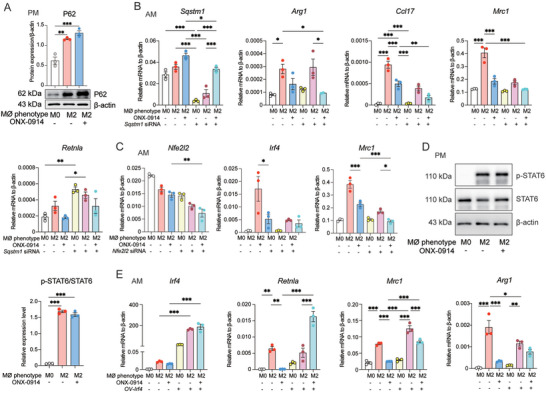
ONX‐0914 Suppresses M2 Polarization via IRF4 Signaling Pathway. A) After pre‐treated with 0.2 µM ONX‐0914 for 6 h and ONX‐0914 administration, the expression of P62 was analyzed by Western blotting in M2 polarization of PM (n = 3). B) In M2‐polarized AMs, after silencing the expression of P62 with siRNA and treating with 0.2 µM ONX‐0914, the expression of M2 marker genes (*Arg1*, *Ccl17*, *Mrc1*, and *Retnla*) was detected by RT‐qPCR (n = 3). C) After silencing the expression of NRF2 with siRNA and treating M2‐polarized AMs with 0.2 µM ONX‐0914, the expression of *Nfe2l2*, and M2 marker genes (*Irf4*, and *Mrc1*) were detected by RT‐qPCR (n = 3). D) After pre‐treated with 0.2 µM ONX‐0914 for 6 h and challenge with ONX‐0914, the expression of STAT6 and p‐STAT6 were detected and analyzed by Western blotting in M2 polarization of PMs (n = 3). E) In M2‐polarized PMs, after the overexpression of IRF4 with plasmid and treating with 0.2 µM ONX‐0914, the M2 marker genes (*Irf4*, *Arg1*, *Ccl17*, and *Mrc1*) were detected by RT‐qPCR(n = 3). F) After the overexpression of IRF4 with plasmid and pretreatment of 0.2 µM ONX‐0914 for 6 h and followed by treating with IL‐4 induced M2 polarization of AMs for 24 h, the M2 marker genes (*Irf4*, *Arg1*, *Ccl17*) were detected by RT‐qPCR (n = 3). *p < 0.05, **p < 0.01, ***p < 0.001.

According to above results, P62 is highly expressed in macrophages treated with ONX‐0914 and plays a pivotal role in the ONX‐0914 suppression of M1 macrophages. As such, we hypothesized that P62 might also contribute to the inhibitory effect of ONX‐0914 on M2 polarization. We conducted a Western blotting to assess P62 expression levels in M2 macrophages post‐treatment with ONX‐0914, and the results demonstrated a notable upregulation of P62 expression in AMs (**Figure** **7**A). To explore whether P62 has a similar regulatory role in M2 under ONX‐0194 treated M1 polarized macrophages, we utilized siRNA to silence the expression of P62. We found that P62 siRNA did not reverse the suppression of M2 marker genes (*Arg1*, *Irf4*, *Ccl17*, *Mrc1* and *Retnla*) by ONX‐0914 in AMs as it did in M1. There was no consistent regulatory effect on M2 (Figure 7B). Additionally, we investigated whether ONX‐0914 inhibits M2 polarization via the NRF2 pathway. After knockdown with Nfe2l2 siRNA, the suppression of M2 markers (*Irf4* and *Mrc1*) became more pronounced (Figure 7C), suggesting that ONX‐0914 did not modulate the inhibition of M2 through NRF2. Notably, the signal transducer and activator of transcription 6 (STAT6) is the key downstream TF of IL‐4 signaling to drive macrophage M2 polarization.^[^ ^31^, ^32^, ^33^ ^]^ Hence, we assessed the activation of STAT6 pretreated with ONX‐0914 by Western blotting analysis. The results showed that ONX‐0914 had no impact on the levels of STAT6 and its phosphorylated form, p‐STAT6, indicating that ONX‐0914 inhibition of M2 polarization was not through the canonical STAT6 signaling pathway (Figure 7D).

A number of studies have reported that interferon regulatory factor 4 (IRF4), a transcription factor, plays a vital role in promoting M2 polarization.^[^ ^34^ ^]^ We overexpressed IRF4 using a plasmid and found that the suppressed M2 marker genes by ONX‐0194 treatment were significantly recovery by overexpressing IRF4, which indicated that IRF4 was involved in the suppression of M2 polarization by ONX‐0914 (Figure 7E).

In summary, these results suggested that ONX‐0914 inhibites M2 macrophage polarization is not through P62, NRF2 and STAT6, rather required the down regulation of IRF4 by ONX‐0914.

### 2.7 NanoONX‐0914 Provides Potential Therapeutic Avenue for COPD by Targeting Immunoproteasome in Polarized Macrophages

**Figure 8:**
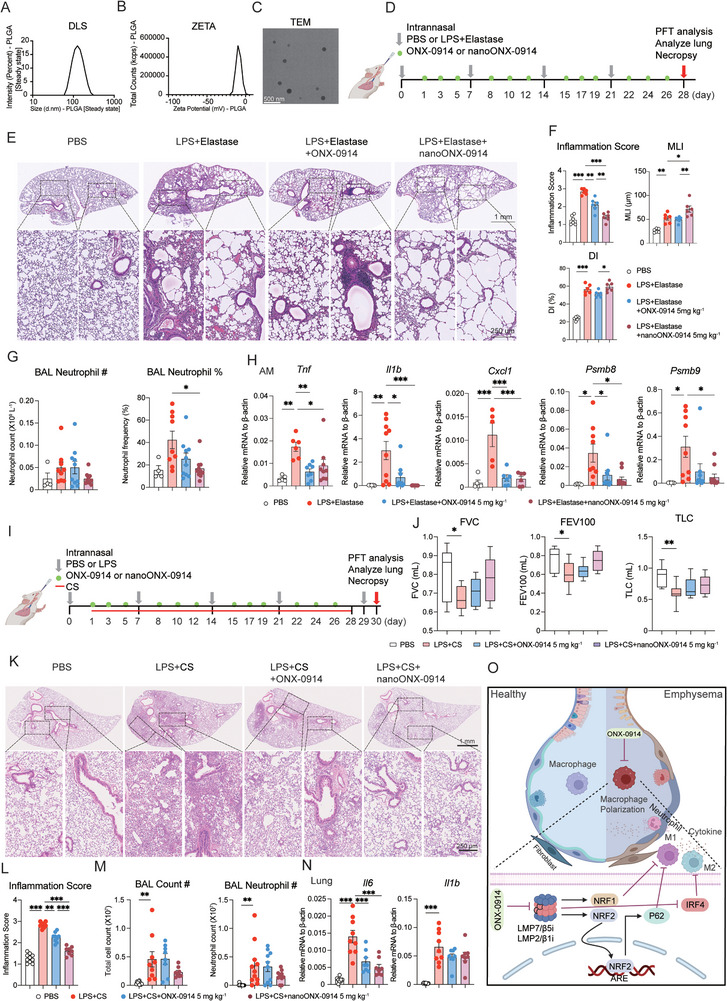
Nanoparticle‐Mediated Delivery of ONX‐0914 to Macrophages Shows Therapeutic Potential for Emphysema. A) Dynamic Light Scattering (DLS) analysis of the encapsulated ONX‐0914 nanoparticles. B) ZETA potential of the encapsulated ONX‐0914 nanoparticles. C) Transmission Electron Microscopy (TEM) image of the encapsulated ONX‐0914 nanoparticles. D) Experimental setup for COPD experiment. ONX‐0914 and nanoONX‐0914 were intranasally administered one day post‐LPS + Elastase challenge, three times weekly for four weeks to induce COPD and n ≥ 6 for each group. E) H&E staining observed the changes of in lung tissue of each group. F) The inflammation and structural destruction of lung tissues were statistically analyzed with inflammation score, mean linear intercept (MLI) and destruction index (DI). G) Neutrophil count and neutrophil frequency in BAL fluid detected in each group using Sysmex hematology analyzer (n ≥ 4). H) Relative gene expression of M1 marker genes (*Tnf, Il1b, Cxcl1*)*, Psmb8, and Psmb9* in AMs of each group by RT‐qPCR (n ≥ 4). I) Experimental setup for LPS + CS mouse models. ONX‐0914 and nanoONX‐0914 were intranasally administered one day post‐LPS challenge, three times weekly for four weeks to induce COPD and each group contains at least 6 mice. J) Pulmonary function indexes, including FVC, FEV100 and TLC, were recorded in each group using PFT Pulmonary Maneuvers. K) Representative images of H&E staining displayed the alveolar changes in lung tissue in each group. L) The inflammation of lung tissue was statistically analyzed by inflammation score. M) Cellular infiltration in BAL fluid was accessed in each group by flow cytometry analysis. N) RT‐qPCR analysis detected M1 marker genes (*Nos2*, *Il1b*, *Il12b*, *Il6* and *Tnf*) in lung tissue of each group. Data was collected from 8 independent mouse. O) The schematic diagram shows that ONX‐0914 targets immunoproteasome subunits LMP7 and LMP2 in macrophages to suppress M1 polarization via activating NRF1 and NRF2‐P62 axis, while the suppression of M2 polarization is regulated by inhibiting IRF4, which ultimately ameliorates experimental emphysema. *p < 0.05, **p < 0.01, ***p < 0.001.

To conclusively establish ONX‐0914′s efficacy in alleviating emphysema airway inflammation by targeting macrophages, we employed a sophisticated approach: encapsulating ONX‐0914 in PLGA nanoparticles (NPs) tailored for macrophage targeting (**Figure** **8**A‐C). The intranasal administration of these nano‐drugs was initiated in our mouse model (Figure 7D). H&E staining unequivocally demonstrated that intranasal administration of the nano‐drug significantly reduced the inflammatory response induced by LPS + Elastase (Figure 8E and F). To further assess the inhibitory effect of nanoONX‐0914 on macrophages infiltration in emphysema mouse models, we performed IF staining on lung tissues from each group using the macrophage‐specific antibody Galectin‐3 and neutrophil‐specific antibody Ly6G, respectively. The results showed that both ONX‐0914 and nanoONX‐0914 effectively reduced infiltration of macrophage and neutrophil in the model, whereas ONX‐914 nano‐drug showed better effect than naked counterpart (Figure S5A,B, Supporting Information). Subsequent analysis revealed a marked reduction both in the frequency and numbers of BAL neutrophils in the experimental emphysema mouse model treated with the nanoONX‐0914 (Figure 8G). Furthermore, RT‐qPCR analysis of AMs in BAL fluid indicated a significant decrease in the expression of M1 (*Tnf*, *Il1b*, and *Cxcl1*), M2 (*Irf4*, *Ccl17*, *Mrc1* and *Retnla*) marker genes and immunoproteasome subunits (*Psmb8 and Psmb9*) upon treatment with both the nano‐drug and naked ONX‐0194 (Figure 8H; Figure S5C, Supporting Information). These compelling results underscored the potential of targeting immunoproteasomes in macrophages using ONX‐0914 nanomedicine as a promising therapeutic approach for emphysema.

Of note, Cigarette smoke (CS) is the leading risk factor to cause COPD. To better simulate the pathogenesis of human COPD, we have established a mouse model using LPS combined with CS to examine the anti‐inflammatory effect of ONX‐0914. In briefly, mice were challenged with nasal instillation of LPS (5 µg µL^−1^) on the day 1 and day 29. From the day 2 to day 30 (excluding the day 29), mice were subjected to CS exposure by placing them in a smoking chamber, with 10 cigarettes/time for 30 min, 5 days/week for a total of 4 weeks. Meanwhile, to seek the potential therapeutic treatment, ONX‐0914 and nanoONX‐0914 (a nano‐delivery system for ONX‐0914 targeted to macrophage) were intranasally administered one day post‐LPS challenge, three times weekly for four weeks (Figure 8I). Pulmonary function tests revealed that both nanoONX‐0914 and ONX‐0914 had an improving effect, with the nanoONX‐0914 demonstrating better results (Figure 8J). Additionally, H&E staining and spectral flow cytometry analysis also showed that the inflammatory cells in both drug treatment groups were significantly reduced compared to the COPD model group, with the nano‐drug showing the most pronounced effect (Figure 8K‐M). In addition, RT‐qPCR analysis showed that ONX‐0914 significantly dampened the expression of M1 (*Il6* and *Il1b*) and M2 (*Arg1*, *Ccl17*, *Mrc1*, and *Irf4*) marker genes, while exerting inconsistent effect on M2 genes in lung tissue (Figure 8N; Figure S5D, Supporting Information). In conclusion, specifically targeting immunoproteasome in macrophages can alleviate LPS + CS induced COPD. ONX‐0914 targeting immunoproteasome to inhibit M1 polarization through NRF1 and NRF2‐P62 aix, while the suppression of M2 polarization is regulated by inhibiting IRF4 (Figure 8O).

Lastly, to explore whether other proteasome inhibitors have similar effect as ONX‐0914 in COPD. Bortezomib (BTZ) is a proteasome inhibitor that was initially approved for the treatment of multiple myeloma (MM).^[^ ^35^ ^]^ In recent years, researchers have begun to explore its potential applications in the treatment of respiratory diseases. BTZ has been found to inhibit the activity of fibroblasts and reduce pulmonary inflammation in a model of pulmonary fibrosis.^[^ ^35^ ^]^ However, its role in other respiratory diseases including COPD and lung cancer has not been well investigated. Here, we have applied BTZ in LPS+CS mouse model in vivo (Figure S6A, Supporting Information) and in polarized M1 and M2 macrophages in vitro to demonstrate whether other proteasome inhibitors have the similar effect as to ONX‐0914. H&E staining results indicated that the administration of BTZ at a dosage of 0.5 mg kg^−1^ showed slight therapeutic effect on ameliorating pulmonary inflammation (Figure S6B,C, Supporting Information). Additionally, RT‐qPCR results revealed that the M1 marker did not show a notable decrease compared to the model group, and the M2 marker did not exhibit consistent changes in comparison to ONX‐0914 in lung tissue (Figure S6D,E, Supporting Information). Furthermore, we compared the inhibitory effect of BTZ with ONX‐0914 in vitro. RT‐qPCR analysis indicated that the inhibitory effect of BTZ on both M1 and M2 polarization were not consistent compared to ONX‐0914 (Figure S6F,G, Supporting Information).

In summary, these results suggested that ONX‐0914 is a potential drug to the therapeutic intervention of COPD by targeting immunoproteasome in macrophage.

## 3 Discussion

Our study delves into the pivotal role of immunoproteasomes within the immune system, elucidating their significant contribution to the pathogenesis of inflammatory diseases, particularly in the context of emphysema. We have demonstrated a robust upregulation of immunoproteasomes in polarized macrophages during the development of experimental emphysema. Crucially, our findings provide compelling evidence that targeting immunoproteasome activity using ONX‐0914 yields substantial therapeutic benefits, including improved lung function and alleviation of lung inflammatory responses, achieved through macrophage depolarization. The intricate molecular mechanisms underlying ONX‐0194‐mediated polarization suppression have been unraveled in our study. We have delineated a pathway involving the activation of transcriptional factors Nrf1 and Nrf2, along with their downstream target P62, highlighting the complex regulatory network at play in ONX‐0194‐mediated M1 polarization suppression. Additionally, ONX‐0914 regulated M2 macrophage depolarization through inhibiting IRF4. These discoveries underscore the immunoproteasome machinery within macrophages as a promising drug target for COPD treatment.

Our study began by successfully establishing an experimental mouse model of emphysema through the intranasal administration of LPS + Elastase to the mouse lung. In this model, we observed an increase in the expression of immunoproteasome subunits LMP2 and LMP7, both in lung tissue and in isolated alveolar macrophages of mice with emphysema. This increase was accompanied by elevated expression of M1 and M2 marker genes, such as the inflammatory cytokines TNF, IL‐1b, IRF4, and ARG1. It's worth noting that immunoproteasome expression can be induced by inflammatory stimuli like TNF, IFN‐γ and LPS.^[^ ^36^ ^]^ Therefore, our findings of increased immunoproteasome levels during emphysema are consistent with the expected response to inflammatory conditions. However, it's important to acknowledge that there have been contrasting reports in the literature regarding immunoproteasome activity in the context of COPD. For instance, Kammerl et al. reported a down‐regulation of immunoproteasome activity in BAL cells and isolated AMs from patients with COPD.^[^ ^37^ ^]^ On the other hand, another study by the same group suggested that immunoproteasome expression and activity were induced in peripheral blood mononuclear cells (PBMCs) from COPD patients.^[^ ^19^ ^]^ This induction was found to correlate significantly with reduced lung function parameters in COPD patients,^[^ ^19^ ^]^ suggesting that immunoproteasome modulation could be a promising therapeutic strategy for COPD intervention.

Indeed, prior to our study, there was a lack of research demonstrating the therapeutic potential of immunoproteasome inhibition in COPD. To address this gap, we investigated the impact of immunoproteasome inhibition using ONX‐0914 in an emphysema mouse model. Our findings revealed that ONX‐0914 significantly reduced the lung inflammatory response in emphysema mice and improved lung function. Notably, while ONX‐0914 had a positive effect on lung inflammation and function, it did not appear to improve the enlargement of the airspace in the lung. Monocytes, as critical components of the innate immune system, can migrate to sites of inflammation and differentiate into macrophages under inflammatory stimulation.^[^ ^38^ ^]^ The role of immunoproteasome inhibition as a therapeutic approach for inflammatory diseases involving macrophage‐mediated immune responses was first demonstrated by the Marcus Groettrup laboratory in 2009.^[^ ^17^ ^]^ Their work showed that selective inhibition of LMP7 by ONX‐0194 could block cytokine production and attenuate the progression of experimental arthritis.^[^ ^17^ ^]^ This inhibition occurred by reducing the production of interleukin‐23 (IL‐23) by activated monocytes and interferon‐gamma and IL‐2 by T cells.^[^ ^17^ ^]^ Another significant piece of evidence came from a study by Nadine Althof and colleagues in 2018.^[^ ^39^ ^]^ They reported that ONX‐0914 treatment could reduce severe virus‐mediated inflammation of the heart by inhibiting the infiltration of monocytes/macrophages into the heart and reducing pro‐inflammatory cytokine and chemokine production.^[^ ^39^ ^]^ Macrophages are central players in the clearance of exogenous particles and the defense against respiratory tract pathogens.^[^ ^40^ ^]^ They are also recognized as important mediators of the inflammatory response in COPD.^[^ ^41^ ^]^ Júlia Benini Kohler et al. reported a close relationship between M1 macrophage polarization and the development of elastase‐induced emphysema, and both M1 and M2 polarizations are associated with COPD patients and CS exposure.^[^ ^42^ ^]^ Our study found that ONX‐0914 treatment significantly inhibited macrophage polarization, which suggests that ONX‐0914 alleviates the inflammatory response in emphysema at least partially by suppressing macrophage polarization. It's worth noting that there might be differences between the effects of genetic knockout of immunoproteasome subunits and pharmacological inhibition. The previous findings and those of Annegret Bitzer et al. also suggested that genetic knockout of LMP2 and LMP7 did not influence PM M1 polarization or iKBa degradation.^[^ ^43^ ^]^ In addition, we reported that genetic knockout of LMP7 enhanced alveolar macrophage M2 polarization by augmenting IL‐4 receptor α (IL4Rα) signaling in 2016.^[^ ^16^ ^]^ These differences may involve distinct regulatory mechanisms that warrant further investigation.

It's intriguing to observe the complex interplay between proteostasis pathways, particularly the ubiquitin‐proteasome system (UPS), unfolded protein response (UPR), and autophagy, in the context of immunoproteasome inhibition by ONX‐0914. Proteostasis, the cellular machinery responsible for maintaining protein homeostasis, is essential for proper cell function and survival.^[^ ^44^, ^45^ ^]^ Proteasome inhibition, as induced by ONX‐0914, leads to the accumulation of ubiquitinated proteins, triggering a compensatory response that includes the unfolded protein response (UPR) and autophagy. The UPR is activated to relieve endoplasmic reticulum (ER) stress caused by the accumulation of misfolded proteins.^[^ ^46^ ^]^ Simultaneously, autophagy, a cellular recycling process, is initiated to remove damaged cellular components and maintain cellular homeostasis.^[^ ^47^ ^]^ Surprisingly, despite the activation of both autophagy and ER stress by ONX‐0914 in M0 and M1 polarized macrophages, blocking these pathways with chloroquine (CQ) and 4‐phenylbutyric acid (4‐PBA) did not rescue M1 polarization. Instead, CQ enhanced ONX‐0914′s inhibitory effect on M1 polarization. This observation suggests a more intricate relationship between immunoproteasome inhibition, autophagy, and ER stress in macrophage polarization. Furthermore, the enhanced inhibitory effect of ONX‐0914 and CQ cotreatment on M1. Similarly, autophagy was activated in M2 macrophages treated with ONX‐0914, which did not affect the suppression of M2 polarization. Polarization might be related to reduce macrophage viability, indicating a potential synergy between proteasome inhibition and autophagy blockade. Interestingly, similar synergistic approaches involving proteasome and autophagy inhibition have been explored in cancer treatment,^[^ ^48^ ^]^ underscoring the importance of understanding these complex interactions for therapeutic interventions.

In our study, we observed that ONX‐0194 stimulation led to an increase in the expression of the autophagy key regulatory molecule P62. P62, a multifunctional scaffold protein, acts not only as a ligand for ubiquitin‐modified proteins but also as a substrate for autophagy and UPS‐mediated degradation.^[^ ^49^ ^]^ Several studies have highlighted the anti‐inflammatory role of P62 in macrophage activation. For instance, Inna S Afonina et al. reported that the deletion of P62 can enhance NLRP3 inflammasome activation and IL‐1β secretion from macrophages in an autophagy‐dependent manner.^[^ ^50^ ^]^ Additionally, Ismail Sergin et al. demonstrated that P62 deficiency exacerbates atherogenic lipid‐induced apoptosis and inflammatory IL‐1β secretion in macrophages.^[^ ^51^ ^]^ In our research, we found that P62 is crucial for the ONX‐0914‐mediated suppression of LPS/IFNγ and CSE‐induced M1 polarization, but not for the ONX‐0914‐mediated suppression of M2 polarization. Notably, this effect was not dependent on the later stages of the autophagy process, as pharmacological blocking with CQ failed to impair ONX‐0914‐induced polarized suppression. Interestingly, it is worth mentioning that the role of P62 in immune regulation appears to be context‐dependent and cell type‐specific. Paradoxically, Zhang et al. demonstrated that P62 overexpression activates the NF‐κB pathway, leading to the increased expression of pro‐inflammatory cytokines and chemokines in keratinocytes,^[^ ^52^ ^]^ suggesting that the function of P62 in regulating immune responses varies based on the specific cellular context and conditions. According to our results, p62 is not necessarily dependent on activation of autophagy to execute the suppressive roles of p62 in the condition of the ONX‐0194 mediated M1 inhibition. We could get an reasonable explanation from previous studies. In 2010, Takeshi Into reported that P62 inhibit formation of the MyD88‐TRAF6 complex and have a suppressive effect on TLR ligand‐induced expression of IL‐6 and NOS2 in macrophages.^[^ ^53^ ^]^ In 2019, Mi‐Jeong Kim. et al reported that the interaction of p62 with TRAF6 inhibited the interaction of ECSIT and TRAF6, resulting in the inhibition of NF‐κB activation needed to produce pro‐inflammatory cytokines by TLR4.^[^ ^54^ ^]^ Therefore, we speculate that interaction of p62 with TRAF6 is the underlying mechanism for the suppressive roles of p62 in this study.

In our final analysis, we employed ATAC‐seq footprinting analysis to identify the upstream transcription factors (TFs) responsible for the regulation of P62. Our results revealed that ONX‐0914 treatment activated both Nrf1 and Nrf2 in M1‐polarized macrophages. Subsequent analysis predicted that only Nrf2 had the potential to bind to the regulatory site of the P62 gene. Our RNA interference (RNAi) experiments confirmed the necessity of Nrf2 for ONX‐0914‐induced upregulation of P62. It is well‐established that proteasomes play a vital role in maintaining cellular homeostasis during oxidative stress.^[^ ^55^ ^]^ Inhibition of proteasome activity, as observed with compounds like bortezomib or MG132, can rapidly induce oxidative stress.^[^ ^45^ ^]^ This, in turn, triggers Nrf2, the master regulator of anti‐oxidative responses, to translocate from the cytoplasm into the nucleus.^[^ ^55^, ^56^ ^]^ There, it binds to antioxidant response elements, initiating the expression of antioxidant genes, including P62. For example, Irene Riz et al. reported that carfilzomib‐mediated proteasome inhibition induces P62 upregulation by activating Nrf2.^[^ ^57^ ^]^ Our study is the first to report that immunoproteasome inhibition by ONX‐0914 can induce the upregulation of P62 via Nrf2 in macrophages. This suggests a common mechanism for the activation of the Nrf2‐P62 axis following both proteasome and immunoproteasome inhibition. Numerous studies have demonstrated that Nrf2 activation is sufficient to suppress M1 macrophage polarization, reduce inflammation, and alleviate oxidative stress in inflammatory diseases.^[^ ^58^, ^59^ ^]^ Therefore, we propose that the Nrf2‐P62 axis contributes to ONX‐0914‐mediated suppression of M1 polarization. As we expected, Nrf2 siRNA effectively rescued the expression of M1 marker genes under ONX‐0914 inhibition. In addition, NRF2 siRNA mediated recovery of M1 marker genes were repress again by P62 over expression under the condition of ONX‐0914 treatment. There functional rescue experiment strongly supported the inhibitory role of Nrf2‐P62 axis upon immunoproteasome inhibition during M1 polarization. This axis was further validated by CUT&Tag and CUT&Tag‐qPCR, it was confirmed that there is a direct binding of Nrf2 to the enhancer and promoter region of the *Sqstm1* gene in ONX‐0914 treated M1 macrophages. This is in line with a study by Viacheslav Mylka et al., where they found that Compound A (CpdA), a selective glucocorticoid receptor modulator, effectively blocked proinflammatory gene expression in macrophages by activating the Nrf2‐P62 axis.^[^ ^60^ ^]^ They also demonstrated that inhibiting the late stages of autophagy with chloroquine (CQ) did not impair CpdA's ability to suppress LPS‐induced inflammatory markers.^[^ ^60^ ^]^ Instead, it enhanced the inhibitory effect of CpdA on IL‐6 secretion.^[^ ^60^ ^]^ We speculate that CQ‐mediated inhibition of autophagy could block the degradation of unwanted proteins, thereby exacerbating oxidative and anti‐oxidative responses, leading to Nrf2 activation and P62 accumulation. Furthermore, a positive feedback loop between P62 and Nrf2 has been described by Ashish Jain et al.^[^ ^61^ ^]^ They showed that P62 uses its KIR domain to stabilize Nrf2, creating a positive feedback loop that contributes to the activation of Nrf2 target genes in response to oxidative stress.^[^ ^61^ ^]^ Masaaki Komatsu et al. also reported that P62 can stabilize NRF2 through competing with the interaction between Nrf2 and Keap1 in 2010. Thus, we also speculate that ONX‐0914 mediated M1 suppression may be regulated by p62 stabilizing NRF2.^[^ ^62^ ^]^ We will include this question in our future study plan. Finally, a recent study by Eilise M. Ryan et al. demonstrated that selective activation of Nrf2 presents an attractive therapeutic approach for alleviating aberrant airway inflammation in COPD by restoring macrophage function.^[^ ^63^ ^]^ This supports our application of ONX‐0194 for COPD treatment.

It is essential to note that Nrf1, synthesized as an endoplasmic reticulum (ER) membrane protein, plays a crucial role in compensating proteotoxic stress, such as proteasome inhibition, by promoting the transcription of proteasome subunit genes.^[^ ^64^, ^65^ ^]^ This action results in the restoration of normal proteasome functions. Consistent with previous findings, our RNA‐seq analysis demonstrated that immunoproteasome inhibition significantly increased the expression of several constitutive proteasome subunit genes, albeit not immunoproteasome subunits. While Nrf2 has been extensively studied, Nrf1, although less reported, has also been shown to negatively regulate M1 polarization.^[^ ^66^ ^]^ Interestingly, our data revealed that silencing both Nrf2 and P62 could only partially recover the expression of M1 marker genes suppressed by ONX‐0914. This observation prompted us to investigate the functional role of Nrf1 in regulating M1 polarization under ONX‐0194 stimulation. Intriguingly, silencing Nrf1 expression also partially rescued the ONX‐0914‐induced suppression of M1 marker gene expression. To our knowledge, there are no existing reports on the role of Nrf1 in the pathogenesis of COPD.

Interestingly, M2 macrophages pretreated with ONX‐0914 also highly expressed P62, but the inhibition of M2 polarization didn't depend on it. Furthermore, we interfered with NRF2 and found that it was not involved in the regulation of M2 by ONX‐0914 either. Of note, both of IRF4 and STAT6 are the key TF to drive M2 polarization. In this study, we found that ONX‐0914 treatment did not impact the expression and activation STAT6, whereas ONX‐0914 can significantly inhibit the IRF4 transcription. In addition, over expression of IRF4 was found to significantly recovery the suppression of M2 polarization by ONX‐0914. However, the specific mechanisms underlying the inhibition of IRF4 by ONX‐0914 during M2 polarization are not fully explored and warrant further in‐depth study.

In recent years, nano‐drug delivery for respiratory systems for have attracted increasing attention due to their advantages, such as improved bioavailability, high drug loading, low toxicity and side effects, good targeting and controlled drug release.^[^ ^67^ ^]^ Macrophages, a phagocyte, can uptake nano‐drugs, and thereby it can be more easily targeted by nano‐drug. In our study, compared to naked ONX‐0914, H&E and macrophage marker immunofluorescence staining unequivocally demonstrated that nano‐drug reduced the inflammatory response induced by LPS + Elastase better than naked drug. In addition, we showed that nano‐ONX‐0194 have better therapeutic effect on lung function, inflammation and immune cell infiltration in CS+LPS induced COPD model. In addition, nano‐drug investigation provided a solid evidence to therapeutic effect of ONX‐0914 in COPD by targeting immunoproteasome in macrophage but not through other cell types.

## 4 Conclusion

In summary, our study demonstrates that immunoproteasome inhibition effectively prevents the development of emphysema by modulating macrophage activation. We have uncovered a novel mechanism by which ONX‐0194 mitigates macrophage inflammation, involving the activation of the NRF1 and NRF2‐P62 axis in M1 polarization, and regulation of IRF4 in M2 polarization. These findings strongly suggest that targeting the immunoproteasome in macrophages holds promise as a therapeutic approach for COPD.

## 5 Experimental Section

### Animals

Specific pathogen‐free (SPF) C57BL/6JGpt mice (male, 7‐week‐old) were procured from Guangdong Yaokang Biotechnology Co., LTD, P.R. China. They were housed in a controlled environment with a relative humidity of 50 ± 20%, temperature set at 22 ± 1 °C, and a 12‐h light/dark reverse cycle. Mice had access to commercial rodent diets and sterilized tap water ad libitum. Animals aged between eight to twelve weeks were euthanized for the collection of lung bronchoalveolar lavage (BAL) and peritoneal macrophages (PMs). All animal experiments were performed according to procedures approved by the Laboratory Animal Ethics Committee of Shenzhen People's Hospital (AUP‐220516‐CSZ‐0351‐01), The Second Clinical Medical College, Jinan University, The First Affiliated Hospital of Southern University of Science and Technology). Every effort was made to minimize animal suffering and reduce the total number of animals used in the study.

### Macrophage Cell Line Culturing

The murine macrophage cell line RAW 264.7 was procured from the American Type Culture Collection (Manassas, VA, USA) and used within the first ten passages. RAW 264.7 cells were cultured in 75‐cm^2^ cell culture plates in Dulbecco's Modified Eagle Medium (DMEM) with high glucose (4.5 g L^−1^, 11965092, Gibco, Thermal Fisher, Massachusetts, USA), supplemented with 10% fetal bovine serum (FBS, P30‐3601, PAN‐Biotech, Aidenbach, Germany), and 100 U mL^−1^ penicillin along with 100 µg mL^−1^ streptomycin (T1300‐100, Solarbio, Beijing, China). The cells were maintained at 37 °C in a humidified atmosphere with 5% carbon dioxide.

### Preparation of Nanoparticle‐capsulated ONX‐0914

The delivery system of ONX‐0914 capsulated with poly(lactic‐co‐glycolic acid) (PLGA) was obtained as follows: 40 mg PLGA and 4 mg ONX‐0914 were homogenized in 2 mL of acetone: dichloromethane (1:4) solution, subsequently were added into 4 mL of polyvinyl alcohol (PVA) aqueous solution (20 mg mL^−1^) and then sonicated for 20 min with 70% power. The homogeneous emulsion was centrifuged at 20000 × *g* for 40 min and washed twice with PBS. The final dispersion was resuspended in Milli‐Q water and stored at 4 °C for further use. The size and parameters of nanoparticle‐capsulated ONX‐0914 was accessed and measured the hydrodynamic diameter and zeta potential by a dynamic light scatter (DLS; Nano‐Zen 3600, Malvern Instruments, UK).

### Primary Cultivation and Treatment of Alveolar Macrophages (AMs) and Peritoneal Macrophages (PMs) In Vitro

Primary murine AMs were isolated and purified from lung bronchoalveolar lavage (BAL) of eight‐week‐old mice. Briefly, mice were anesthetized by intraperitoneal (i.p.) injection of avertin solution (250 mg kg^−1^, a mixture of 2,2,2‐tribromoethanol (Sigma‐Aldrich, #T48402‐5GM) with 2‐methyl‐2‐butanol, tert‐amyl alcohol 99+% (Sigma‐Aldrich, #240486‐5ML). The trachea was exposed and cannulated with a 20‐G catheter, and lavages were flushed out with 1 mL of cold and sterile PBS, repeated 7–8 times. After centrifugation at 1300 rpm for 15 min at 4 °C, the BAL supernatant was discarded, and the cell pellets were resuspended in pre‐warmed DMEM/high glucose supplemented with 10% FBS and 100 U mL^−1^ penicillin plus 100 µg mL^−1^ streptomycin. Alternatively, cells were resuspended in pre‐cold PBS for spectral flow cytometry analysis.

Prior to isolation, mice were pre‐injected with pentobarbital and 3% thioglycolate (Fisher Scientific, R08852, Pennsylvania, USA) for 72 h. After the pre‐injection period, 8‐week‐old mice were euthanized by CO_2_ inhalation. Residential PMs were harvested from peritoneal lavage fluid by flushing the peritoneum 5–6 times with 5 mL of cold sterile PBS using a syringe. The subsequent procedures were carried out following the same protocol used for alveolar macrophages (AMs). Finally, the isolated cells were counted and seeded at a density of 2 × 10^^5^ cells per well in a volume of 200 µL in 48‐well plate (or 5 × 10^^4^ cells per well in 100 µL of 96‐well plate) for further treatments.

### Polarization of Alveolar Macrophages (AMs) and Peritoneal Macrophages (PMs) Ex Vivo

Residential AMs and PMs isolated from naïve mice were plated in 96‐well tissue culture plates and incubated at 37 °C in a humidified atmosphere with 5% carbon dioxide for 4 h. To induce the classical M1 macrophage activation, cells were stimulated with 1 µg mL^−1^ Lipopolysaccharides (LPS, Solarbio, #L8880, Beijing, China) and 20 ng mL^−1^ recombinant mouse interferon‐gamma (IFN‐γ, Genscript Biotech, #Z02916, Nanjing, China) for 24 h. It was induced M2 type macrophages with 20 ng L^−1^ Interleukin‐4 (IL‐4, Genscript Biotech, #P07750, Nanjing, China). These M1 and M2 phenotype AMs or PMs were then prepared for further treatments.

### Administration of ONX‐0914, Chloroquine, and 4‐Phenylbutyric Acid (4‐PBA) In Vitro

To investigate the impact of ONX‐0914 on macrophage autophagy and endoplasmic reticulum stress (ERS) at the protein level, alveolar macrophages (AMs) underwent four different treatment paradigms:1) M0 phenotype AMs were cultured with 0.2 µM ONX‐0914 for varying durations of 0, 6, 12, 18, and 24 h. 2) AMs were pre‐treated with 0.2 µM ONX‐0914 for 6 h, followed by co‐incubation with LPS/IFN‐γ or IL‐4 for an additional 24 h. 3) AMs were pre‐treated with 0.2 µM ONX‐0914 for 6 h, and then co‐incubated with 10 µM chloroquine (CQ) and LPS/IFN‐γ or IL‐4 for 24 h. 4) AMs were pre‐treated with 0.2 µM ONX‐0914 for 6 h, and then co‐incubated with 1 mM 4‐Phenylbutyric acid (4‐PBA) and LPS/IFN‐γ for 24 h. Following the specified time points, whole cell lysates and total mRNA were collected for subsequent experiments.

### Activation and Immunofluorescence of Autophagy and ERS In Vitro

For the assessment of autophagy in AMs, p‐BABE‐puro‐mchermy‐EGFP‐tagged LC3B expression was employed as a reliable indicator. AMs were cultured in a 24‐well plate for a minimum of 2 h. Control vector p‐BABE‐puro‐mchermy‐EGFP‐blank vector (140‐101, cat#CBA‐401) and p‐BABE‐puro‐mchermy‐EGFP‐LC3B expression vector (140102, cat#CBA‐401, Cell Biolabs, Shanghai, China) were introduced into the cells using jetPRIME^@^ transfection reagent (Ref#114‐01, Polyplus, Beijing, China) and cultured for an additional 24 h. Subsequently, AMs were treated with 0.2 µM ONX‐0914 and co‐cultured with the plasmids for 24 h. After treatment, AMs were fixed with 4% paraformaldehyde (PFA, P0099, Beyotime, Shanghai, China) for 30 min at room temperature (RT), followed by three gentle washes with PBS. The cells were permeabilized with 0.3% Triton X‐100 in PBS for 30 min at RT and then incubated with 1% bovine serum albumin (BSA, #A1933, Sigma‐Aldrich) for 1 h at RT. Subsequently, the samples were co‐stained with DAPI for 10 min and mounted using anti‐fade mounting medium (P0126, Beyotime, Shanghai, China). Confocal images were acquired using a confocal laser microscopy system (Leica TCS SP8 X, Leica, Germany).

Besides, the same approach to induced autophagy in PMs was applied. After that, PMs were fixed with 4% paraformaldehyde (PFA, P0099, Beyotime, Shanghai, China) for 30 min at room temperature (RT), followed by three gentle washes with PBS. The cells were permeabilized with 0.3% Triton X‐100 in PBS for 30 min at RT and then incubated with 1% bovine serum albumin (BSA, #A1933, Sigma‐Aldrich) for 1 h at RT. Subsequently, PMs were then incubated overnight at 4 °C with anti‐rabbit LC3B (1:200) (Table 2). The following day, the samples were co‐stained with DAPI for 10 min and mounted using anti‐fade mounting medium (P0126, Beyotime, Shanghai, China). Confocal images were acquired using a confocal laser microscopy system (Leica TCS SP8 X, Leica, Germany).

For the assessment of ER Stress, PB transposase (Pbase)‐mediated integration‐GFP‐KDEL (Pbase‐GFP‐KDEL) expression was employed as a canonical methods. Combination of KDEL and its receptor is regarded as indicator of ERS activation.^[^ ^68^ ^]^ Thus, the expression of KDEL in endoplasmic reticulum was indicated to activation of ERS. Same transfection and activation methods as autophagy plasmid described above. Specifically, GFP‐KDEL plasmid is homogenized with Pbase at a 3:1 ratio, subsequently, jetPRIME^@^ transfection reagent was applied to assist GFP‐KDEL plasmids complex into nucleus.^[^ ^69^ ^]^ The expression of KDEL was observed by immunofluorescence assay as autophagy plasmids described above.

### Library Preparation and Data Analysis of Bulk RNA‐seq and ATAC‐seq

For bulk RNA‐seq analysis, PMs from two to three mice in each group were pooled to obtain 50000 cells for each replicate, and four independent replicates were analyzed for each group. Total RNA was extracted from each sample using the RNA isolater kit (Vazyme, R401‐01, Nanjing, China). Complementary DNA (cDNA) libraries were prepared using the HiScript II Q RT SuperMix kit (Vazyme, R223‐01). After quality control assessment with Qubit and Agilent 5400 system (Agilent, USA), the qualified cDNA libraries were sequenced on Illumina platforms using PE150 sequencing. The Assay for Transposase‐Accessible Chromatin with Sequencing (ATAC‐Seq) was a robust method for evaluating chromatin accessibility across the genome. In brief, AMs cells were cultured in DMEM/high glucose supplemented with 10% FBS until reaching ∼80% confluence. For ATAC‐seq, 100000 cells were lysed in chilled Lysis Buffer, and sequencing libraries were prepared using the Hyperactive ATAC‐Seq Library Prep Kit for Illumina (Vazyme, TD711, Nanjing, China) following the manual protocol. Subsequently, fragmented DNA products were extracted using ATAC DNA Extract Beads. Amplified products were purified using ATAC DNA Clean Beads through a two‐step sorting protocol (0.55×/1.2×). Cleaned‐up libraries were quantified, pooled, and sequenced by Novogene. ATAC‐seq libraries were sequenced on Illumina platforms using PE150 sequencing.

Raw sequence reads underwent quality evaluation using FastQC v0.11.9.^[^ ^70^ ^]^ Trimming of low‐quality bases (<20) and removal of adapter sequences from raw reads was performed using Cutadapt v2.8 before alignment.^[^ ^71^ ^]^ Alignment to the mm10 reference genome was carried out using Bowtie2 v2.2.5 with the option “—very‐sensitive”.^[^ ^72^ ^]^ To ensure data integrity, duplicate sequences were removed using MarkDuplicates v3.0.0 implemented in Picard tools (http://broadinstitute.github.io/picard/). Additionally, sequences originating from mitochondria and blacklist regions were filtered out using Samtools v1.10 for ATAC‐Seq data.^[^ ^73^ ^]^ For gene expression analysis, raw counts were summarized using FeatureCounts v2.0.0^[^ ^74^ ^]^ and imported into R v4.1.0 for further processing.^[^ ^75^ ^]^ The DESeq2 package was utilized for estimating library size factors, normalizing counts, and conducting differential expression analyses.^[^ ^76^ ^]^ Significantly differentially expressed genes (DEGs) were identified based on |log_2_FC| > 1 and false discovery rate (FDR) < 0.05, corrected using the Benjamini‐Hochberg method.^[^ ^77^ ^]^ Functional enrichment analysis was performed using clusterProfiler v3.18.1 for Gene Ontology (GO) enrichment analysis.^[^ ^78^ ^]^ Geneset enrichment analysis (GSEA) was conducted using GSEApy v0.10.4.^[^ ^79^ ^]^ Visualization of results, including heatmap generation, was accomplished using ComplexHeatmap v2.6.2.^[^ ^79^ ^][^ ^80^ ^]^

After alignment, quality control and evaluation of insert size distribution for ATAC‐seq data were conducted using ATACseqQC.^[^ ^81^ ^]^ Peak calling from ATAC‐seq data was performed using macs2 v2.2.9 with specific parameters including “—nomodel,” “—shift 100,” “—extsize 200,” “—broad,” and “—qvalue 0.05.”^[^ ^82^ ^]^ To identify binding sites and motifs, non‐redundant position frequency matrices (PFMs) for vertebrates were obtained from the JASPAR 2022 database.^[^ ^83^ ^]^ TOBIAS v0.16.0 was employed for peak correction, footprint scoring, and binding site tests for all motifs.^[^ ^84^ ^]^ Differential binding sites for Nrf2 and Nrf1 were determined based on a log_2_FC threshold of >2 and an adjusted p‐value cutoff of <0.05. The genes associated with these binding sites were annotated using ChIPseeker v1.34.1.

### Library Preparation and Data Analysis of CUT&Tag

Following the steps outlined in the NovoNGS CUT&Tag 4.0 High‐Sensitivity Kit (for Illuminao) manual (Novoprotein Scientific, #N259‐YH01), the experimental cells were extracted for the open chromatin regions associated with NRF2 binding, and then these regions were subjected to PCR amplification. Subsequently, quality control and sequencing were performed.

The CUT&Tag data analysis began with the preprocessing of the raw sequencing reads, which involved the trimming of the data to eliminate low‐quality sequences and adapters using Trimmomatic v0.39^[^ ^85^ ^]^ with parameters “LEADING: 3 TRAILING: 3 SLIDINGWINDOW: 4:20 MINLEN: 60”. Following this initial cleanup, the high‐quality reads were mapped to the reference genome mm10 employing Bowtie2 v2.2.5. Subsequently, the BAM files were converted into BigWig format using deepTools v3.5.1^[^ ^86^ ^]^ with CPM (counts per million) normalization, allowing for the quantification and visualization of NRF2 binding intensity across the genome. Peak calling was executed using SEACR^[^ ^87^ ^]^ with its default parameters, which efficiently pinpointed the locations of NRF2 interaction with the DNA. The resulting BigWig and peak‐annotated bed files were then visualized using the Integrative Genomics Viewer (IGV),^[^ ^88^ ^]^ providing a comprehensive graphical representation of NRF2 binding patterns and intensities.

### Murine LPS + Elastase Model

Eight‐week‐old SPF healthy C57BL/6JGpt mice were randomly divided into five experimental groups (n = 7 mice per group) and subjected to three different treatment paradigms: 1) Vehicle control group received intranasal administration of PBS. 2) The LPS + Elastase group was intranasally administered with a cocktail of 50 µL containing 7 µg of LPS (Solarbio, #L8880) and 1.2 U of porcine pancreatic elastase (Macklin, #E808882) once a week for 4 weeks. 3) The ONX‐0914 treatment groups received different doses of ONX‐0914 (1.0, 2.5, and 5.0 mg kg^−1^, Selleck, #S7172) intranasally one day after the LPS + Elastase challenge. This treatment was administered three times a week for 4 weeks. Terminal measurements and analyses, including animal necropsy, pulmonary function testing, flow cytometry analysis of lung bronchoalveolar lavage (BAL), and collection of lung tissue, were performed on day 28. The experimental scheme for the mouse model is illustrated in Figure 1A.

### Murine LPS + CS Model

According to published articles,^[^ ^89^, ^90^, ^91^, ^92^ ^]^ C57BL/6JGpt mice (male, 8‐weeks‐old, 10 mice per group) were administered LPS (5 µg µL^−1^; Solarbio, #L8880) intranasally on Day 1 and Day 29. From Day 2 to Day 30 (excluding Day 29), the mice were subjected to cigarette smoke (CS; Hongtashan, Hongta Group, China Tobacco) exposure by placing them in a mouse smoking chamber, with 10 cigarettes per session, for 30 min each session, 5 days a week, for a total duration of 4 weeks.

### Lung Function Measurement

Pulmonary function tests (PFTs) were conducted on 7 mice from each group, excluding those used for sample collection. Lung function was assessed using a BUXCO PFT Controller (DSITM, Harvard Bioscience, USA) following the manufacturer's instructions. Briefly, mice were anesthetized via intraperitoneal injection with 200 mg kg^−1^ of avertin. The trachea was exposed, cannulated with a catheter, and tracheostomized mice were intubated and placed in the body plethysmograph. The average breathing frequency was set to 150 breaths/min. Forced vital capacity (FVC), total lung capacity (TLC), and forced expiratory volume in first 100 milliseconds of exhalation (FEV100), functional residual capacity (FRC), the ratio of the forced expiratory volume in the first 0.1 second of exhalation and the forced vital capacity (FEV0.1/FVC), the residual volume (RV), and peak expiratory flow were recorded during fast flow volume.

### Flow Cytometry Analysis

After lung function assessment, bronchoalveolar lavage (BAL) was performed by washing the lungs with 0.7 mL of flow buffer (PBS, 2% BSA, and 2 mM EDTA) three times in each right lung of the mouse. Total cells in BAL and peripheral blood cells were quantified using a hematology analyzer (XN‐1000, Sysmex, USA). Samples were incubated with anti‐mouse monoclonal CD16/32 (clone 93, #14‐0161‐86, eBioscience, Invitrogen) to block Fc‐γ II/III receptors for 30 min at 4 °C in the dark. Equal numbers of cells (1 million) were then stained in the dark with various antibodies (refer to Table 2) for 30 min at 4 °C. Viable alveolar macrophages (AMs) were identified as CD45^+^SiglecF^+^CD11c^+^, eosinophils (Eos) as CD45^+^SiglecF^+^CD11c^−^, neutrophils (NPs) as CD45^+^SiglecF^−^Ly6G^+^, and monocytes (Mos) as CD45^+^SiglecF^−^CD11b^+^Ly6C^+^. Samples were washed with flow buffer, and flow cytometry was performed using the Cytek Aurora CS full spectrum profiling system (California, USA). Data analysis was conducted using FlowJo software (v10.4.0).

### Lung Histology

After intranasal administration of ONX‐0914 for 4 weeks, COPD mice and control counterparts were deeply anesthetized with avertin (250 mg kg^−1^) and perfused with 20 mL PBS followed by 20 mL of 10% neutral‐buffered formalin through the left cardiac ventricle. The left lung, which had not been lavaged, was collected, fixed in 10% neutral‐buffered formalin overnight, and embedded in paraffin. Tissue blocks were sectioned at 4 µm thickness, and slides were deparaffinized and rehydrated. Hematoxylin and Eosin (H&E) staining was performed using the H&E Kit (Solarbio, # G1120, Beijing, China) following the vendor's guidelines. Images were captured using a Nanozoomer digital slice scanner (Hamamatsu Photonics) and analyzed with NDP.view2 software (Hamamatsu).

### Lung pathological analysis

Lung tissues H&E staining was statistically analyzed. Pulmonary inflammation and emphysema were assessed by inflammation score, destruction index (DI) and mean lining interval (MLI). Three slides were taken from each mouse and three fields were taken from each slide by NDP.view2 Viewing software. Then manual calculation and GraphPad Prism 9.0 were used for statistical analysis.^[^ ^93^, ^94^, ^95^ ^]^

### Lung tissue Immunofluorescence

Lung tissue slides were deparaffinized, rehydrated and antigen retrieval. After that, slides were incubated with 5% normal goat serum (Goat Serum for Blocking, #BL1097A, Biosharp) in PBS for 30 min at RT. Subsequently, slides were then incubated overnight at 4 °C with anti‐rat Glactin3 (1:200, Thermo Fisher Scientific, #14‐5301‐82) (Table 2). On the following day, the primary antibody was rinsed with PBS, and then Goat Anti‐Rat IgG H&L Alexa Fluor 488 (1:500, abcam, # ab150157) was added for 1 h incubation at RT in the dark. Next, the samples were co‐stained with DAPI for 10 min and mounted using anti‐fade mounting medium (P0126, Beyotime, Shanghai, China). Confocal images were acquired using a confocal laser microscopy system (Leica TCS SP8 X, Leica, Germany).

### Western blotting

RAW 264.7 cells, AMs, PMs, and lung tissues from COPD mice were lysed using RIPA buffer supplemented with 1% protease inhibitor cocktail and 1% phosphatase inhibitor on ice for 30 min. Cell debris was removed after centrifugation at 12000 rpm for 30 min. Protein concentration was measured using the standard BCA protein assay kit (Beyotime, #P0011, Shanghai, China). All samples were mixed with sample loading buffer and heated for 15 min at 95 °C. Equal amounts of protein lysates (20 µg) were separated on 12.5% SDS‐PAGE gels and then transferred onto PVDF membranes (Millipore, Billerica, MA, USA). Non‐specific protein binding sites were blocked with 5% BSA (Sigma‐Aldrich, #A1933) for 1 h at room temperature. Membranes were then incubated overnight at 4 °C with primary antibodies (Table 2). The following day, membranes were incubated with HRP‐conjugated anti‐rabbit (1:1000, 7074P2, CST) or anti‐mouse antibodies (1:1000, 7076S, CST) secondary antibodies for 1 h at room temperature. After washing the membranes with 0.1% PBST three times for 5 min each at room temperature, horseradish peroxidase (HRP)‐conjugated goat anti‐mouse secondary antibodies (1:1000; #BA1051, Boster) or anti‐rabbit secondary antibodies (1:1000; #BA1055, Boster) were used to incubate the membranes for 1 h at room temperature. Subsequently, membranes were washed in 0.1% PBST three times for 5 min each at room temperature. The immunoreactive bands were visualized using an enhanced chemiluminescence (ECL) kit (#170‐5061, Bio‐Rad Laboratories) and Chemiluminescence Imaging System (GeneGnome XRQ‐NPC, Syngene, Cambridge, UK). Signal intensities were quantified using ImageJ software.

### In vitro siRNA and/or plasmid Transfection

AMs were cultured in pre‐warmed DMEM/high glucose supplemented with 10% FBS, 100 U mL^−1^ penicillin, and 100 µg mL^−1^ streptomycin. AMs were transfected with short interfering RNAs (siRNAs) or plasmid at a concentration of 100 nM using jetPRIME^@^ transfection reagent (Ref#114‐01, Polyplus) for 12 h. Subsequently, they were co‐cultured with M1 or M2 phenotypic AMs that had been pretreated with 0.2 M ONX‐0914 for 24 h. Additionally, following the manufacturer's instructions for Lipo3000, the plasmid and siRNA into the cells and performed an overnight transfection was co‐transfected, after which a pretreatment with ONX‐0914 and proceeded with macrophage polarization was conducted. Finally, cells were harvested to assess the targeting efficiencies through RT‐qPCR and Western blotting. The plasmids used in this study included p3xFLAG‐P62 var2 (#204576, Addgene) and pMIG‐IRF4 (#58987, Addgene). The siRNA duplexes were synthesized by Genepharma (Shanghai, China), and the sequences of siRNAs were as follows: Sqstm1, 5′‐GAUCUGCGAUGGCUGCAAUTT‐3′, 5′‐AUUGCAGCCAUCGCAGAUCTT‐3′; Nfe2l1, 5′‐GCAUAGACCUGGACAAUUATT‐3′, 5′‐UAAUUGUCCAGGUCUAUGCTT‐3′; Nfe2l2, 5′‐CCGAAUUACAGUGUCUUAATT‐3′, 5′‐UUAAGACACUGUAAUUCGGTT‐3′.

### Real‐time Reverse Transcription‐PCR (RT‐qPCR) Analysis

Total mRNA was extracted from either M0 or M1 phenotype AMs using the RNA isolater kit (Vazyme, R401‐01, Nanjing, China) following the manufacturer's protocol. The concentration of RNA was quantified using a NanoDrop 2000 spectrophotometer (Thermal Fisher Scientific). To synthesize cDNA, the PrimeScript RT reagent Kit (#RR037A, Takara) was used according to the manufacturer's instructions. The primer sequences used for RT‐qPCR are provided in Table 1. RT‐qPCR was carried out on an AB7500 Fast Real‐Time PCR system (Applied Biosystems) using TB Green Premix Ex Taq (#RR420A, Takara). The relative expression of target genes and the housekeeping gene β‐actin were calculated using the 2^^(‐ΔCt)^ method.^[^ ^14^ ^]^

**Table 1:** Primers for RT‐qPCR.

Target gene	Forward primer (5′‐3′)	Reverse primer (5′‐3′)
β‐actin	TCCATCATGAAGTGTGACGT	GAGCAATGATCTTGATCTTCAT
Nos2	CCTGTGAGACCTTTGATG	CCTATATTGCTGTGGCTC
Il1b	CAACCAACAAGTGATATTCTCCATG	GATCCACACTCTCCAGCTGCA
Il12b	GGAAGCACGGCAGCAGAATA	AACTTGAGGGAGAAGTAGGAATG
Il6	TAGTCCTTCCTACCCCAATTTCC	TTGGTCCTTAGCCACTCCTTC
Tnf	CAACCAACAAGTGATATTCTCCATG	GATCCACACTCTCCAGCTGCA
Ccl2	TTAAAAACCTGGATCGGAACCAA	GCATTAGCTTCAGATTTACGGGT
Ccl3	TTCTCTGTACCATGACACTCTGC	CGTGGAATCTTCCGGCTGTAG
Cxcl1	GCTGGGATTCACCTCAAGAA	TCTCCGTTACTTGGGGACAC
Cxcl2	TCCAGAGCTTGAGTGTGACG	TCCAGGTCAGTTAGCCTTGC
Cxcl3	TTCTCTGTACCATGACACTCTGC	CGTGGAATCTTCCGGCTGTAG
Irf4	AAAGGCAAGTTCCGAGAAGGG	CTCGACCAATTCCTCAAAGTCA
Arg1	GGAACCCAGAGAGAGCATGA	TTTTTCCAGCAGACCAGCTT
Ccl17	TTGTGTTCGCCTGTAGTGCATA	CAGGAAGTTGGTGAGCTGGTATA
Mrc1	CATGAGGCTTCTCCTGCTTCT	TTGCCGTCTGAACTGAGATGG
Retnla	CGAGTAAGCACAGGCAGT	CCAGCTAACTATCCCTCCAC
Sqstm1	CCGGCTGATTGAGTCCCTC	CCCCGATGTCGTAATTCTTGG
Nfe2l1	GAGCAGGGATTCGGTGAAGATTT	TCGTCCATCTTGCAGTTCCTTAT
Nfe2l2	TGGCAGAGACATTCCCATTTGT	CTTGCTCCATGTCCTGCTCTATG
Psmb8	ATGGCGTTACTGGATCTGTGC	CGCGGAGAAACTGTAGTGTCC
Psmb9	CATGAACCGAGATGGCTCTAGT	TCATCGTAGAATTTTGGCAGCTC
Ulk1	TGGAGGTGGCCGTCAAATG	CGCATAGTGTGCAGGTAGTC
Gabarapl1	AGGACCACCCCTTCGAGTATC	GCACAAGGTACTTCCTCTTATCC
Gabarapl2	GGAACACAGATGCGTGGAAT	GATGTCCGAGGGGACCAAGTA
Atg3	CTGGAGATCACTTAGTCCACCA	GTCGGAAGATATGCCTTCACTTT
Atg4a	GAAGGAAGTTTTCCCCGATTGG	GGGTTGTTCTTTTTGTCTCTCCC
Atg4b	CGGCACTTAGGTCGAGATTGG	ACTCCCATTTGCGCTATCTGA
Atg5	CACCCCTGAAATGGCATTATCC	TGGACAGTGTAGAAGGTCCTTT
Atg7	TCTGGGAAGCCATAAAGTCAGG	GCGAAGGTCAGGAGCAGAA
Becn1	AGGCGAAACCAGGAGAGAC	CCTCCCCGATCAGAGTGAA
Vps11	AAAAGAGAGACGGTGGCAATC	AGCCCAGTAACGGGATAGTTG
Wipi1	AATCCCTGACGTGTATATCGTGG	GCCGAGGTTTTGTGTGACTGA
Sqstm1 promoter	CGGTCTAGGTACGGACGAAA	TGTGGTCACCCATGTATTCGG
Sqstm1 enhancer	GGCGGGAATAAAGAATCTGGC	CTAGGCAGGTGCTCTGTCAC

**Table 2:** Antibodies and reagents list of Western blotting and FACS.

Antibodies	Source	Identifier
anti‐rabbit ARG1 (1:1000)	Cell Signaling Technology	93 668
anti‐rabbit Bip(1:1000)	Cell Signaling Technology	3177T
anti‐rabbit eIF2α (1:1000)	Cell Signaling Technology	5324T
anti‐rabbit p‐eIF2α (1:1000)	Cell Signaling Technology	3398T
anti‐rabbit IRF4 (1:1000)	Cell Signaling Technology	62 834
anti‐rabbit GADD153 (CHOP) (1:1000)	Cell Signaling Technology	5554
anti‐rabbit IL‐1β (1:1000)	Cell Signaling Technology	31 202
anti‐rabbit JNK1 (1:1000)	Cell Signaling Technology	3708T
anti‐rabbit iNOS (1:1000)	Cell Signaling Technology	2982
anti‐rabbit p‐JNK1 (1:1000)	Cell Signaling Technology	4668T
anti‐rabbit LMP2 (1:1000)	Abcam	ab242061
anti‐rabbit LMP7 (1:1000)	Abcam	Ab3327
anti‐rabbit LC3B (1:1000)	Cell Signaling Technology	2775S
anti‐rabbit STAT6 (1:1000)	Cell Signaling Technology	9362
anti‐rabbit p‐STAT6 (1:1000)	Cell Signaling Technology	56 554
anti‐rabbit P62/SQSTM1 (1:1000)	Sigma‐Aldrich	P0067
anti‐rabbit YM1/2 (1:1000)	Abcam	ab192029
anti‐mouse β‐actin (1:3000)	Sigma‐Aldrich	A5316
HRP‐conjugated anti‐rabbit antibodies (1:3000)	Cell Signaling Technology	7074P2
HRP‐conjugated anti‐mouse antibodies (1:3000)	Cell Signaling Technology	7076S
7‐aminoactinomycin D (7‐AAD, 1:500)	Beyotime	ST515
Rat monoclonal anti‐mouse CD45 APC/Cyanine7 (1:300)	BioLengend	103 116
Rat monoclona anti‐mouse CD170 (Siglec‐F) Brilliant Violet 421 (1:300)	BioLengend	155 509
Rat monoclonal anti‐mouse/human CD11b AF700 (1:300)	BioLengend	101 222
Hamster monoclonal anti‐mouse CD11c APC (1:300)	BioLengend	550 261
Rat monoclonal anti‐mouse Ly‐6G FITC (1:300)	BioLengend	127 605
Rat monoclonal anti‐mouse Ly‐6C PE/Cyanine7 (1:300)	BioLengend	128 018

### Statistical Analysis

Data were analyzed and graphed using GraphPad Prism 9.0 (GraphPad Software Inc.). Statistical differences between groups were assessed using unpaired two‐tailed Student *t*‐tests. One‐way ANOVA followed by Tukey's *post‐hoc* test was used to compare multiple groups. For non‐parametric data, unpaired two‐tailed *Mann‐Whitney* tests were employed. All data were presented as mean ± SEM. A p‐value of ≤ 0.05 was considered statistically significant (ns = no significant difference; *p < 0.05; **p < 0.01; ***p < 0.001).

## Conflict of Interest

The authors declare no conflict of interest.

## Author Contributions

B.G., X.S., and Q.J. contributed equally to this work and should be considered co‐first authors. S.C. contributed to concept and design. B.G., X.S., and Q.J. performed acquisition, analysis, and interpretation of data. B.G., and S.C. drafted the manuscript. B.G., and X.S. performed statistical analysis. Y.P., Y.Y., Y.L., S.C., W.Z., L.W., Y.L., L.R., R.L., H.X., Y.W., X.C., G.H., L.R., and C.Q. provided administrative, technical, or material support. S.C., R.C., and L.W. performed supervision.

## Supporting information

Supporting Information

## Data Availability

The data that support the findings of this study are available from the corresponding author upon reasonable request.
