# Correction to “Targeting Immunoproteasome in Polarized Macrophages Ameliorates Experimental Emphysema via Activating NRF1/2‐P62 Axis and Suppressing IRF4 Transcription”

**DOI:** 10.1002/advs.202524136

**Published:** 2026-01-21

Guo B, Shi X, Jiang Q, et al. Targeting Immunoproteasome in Polarized Macrophages Ameliorates Experimental Emphysema via Activating NRF1/2‐P62 Axis and Suppressing IRF4 Transcription. *Adv Sci (Weinh)*. 2024;11(44):e2405318. https://doi.org/10.1002/advs.202405318.

1. In the “Results” section, the text “Interestingly, Nrf2, but not Nrf1, was identified as binding to the P62 gene regulatory site (Figure 6J,K).” was incorrect. This should have read: “As expected, both Nrf1 and Nrf2 were identified as binding to the P62 gene regulatory site (Figure 6J,K).”
2. In the “Discussion” section, the sentence “Subsequent analysis predicted that only Nrf2 had the potential to bind to the regulatory site of the P62 gene” has been removed.
3. A replacement of the images in Figure 5H, Figures 6B,J, and Figure 7A has been performed with the corresponding final versions.
4. Supplement the missing antibody information of commercial source in Table 2 as follows:

**NRF1**: Anti‐rabbit TCF11/NRF1 (D5B10) (1:1000), Cell Signaling Technology, #8052

**NRF2**: Anti‐rabbit NRF2 (1:500), Cell Signaling Technology, #12721T

We apologize for this error.

**2. Results**

**2.5 ONX‐0914 Suppresses M1 Polarization via NRF1 and NRF2‐P62 Signaling Pathway**

This activation was further supported by increased nuclear localization of NRF2 following ONX‐0194 treatment, as shown by NRF2 antibody‐based fluorescent staining (Figure 6H). RT‐qPCR results also demonstrated that ONX‐0914 enhanced CSE‐stimulated Nrf2 expression in AMs (Figure 6I). Additionally, using Transcription Factor Occupancy By Investigation of ATAC‐seq Signal (TOBIAS) analysis, we predicted the Nrf1 and Nrf2 binding sites in ONX‐0194‐treated M1 macrophages. As expected, both Nrf1 and Nrf2 were identified as binding to the P62 gene regulatory site (Figure 6J,K).

**3 Discussion**

In our final analysis, we employed ATAC‐seq footprinting analysis to identify the upstream transcription factors (TFs) responsible for the regulation of P62. Our results revealed that ONX‐0914 treatment activated both Nrf1 and Nrf2 in M1‐polarized macrophages. Our RNA interference (RNAi) experiments confirmed the necessity of Nrf2 for ONX‐0914‐induced upregulation of P62.


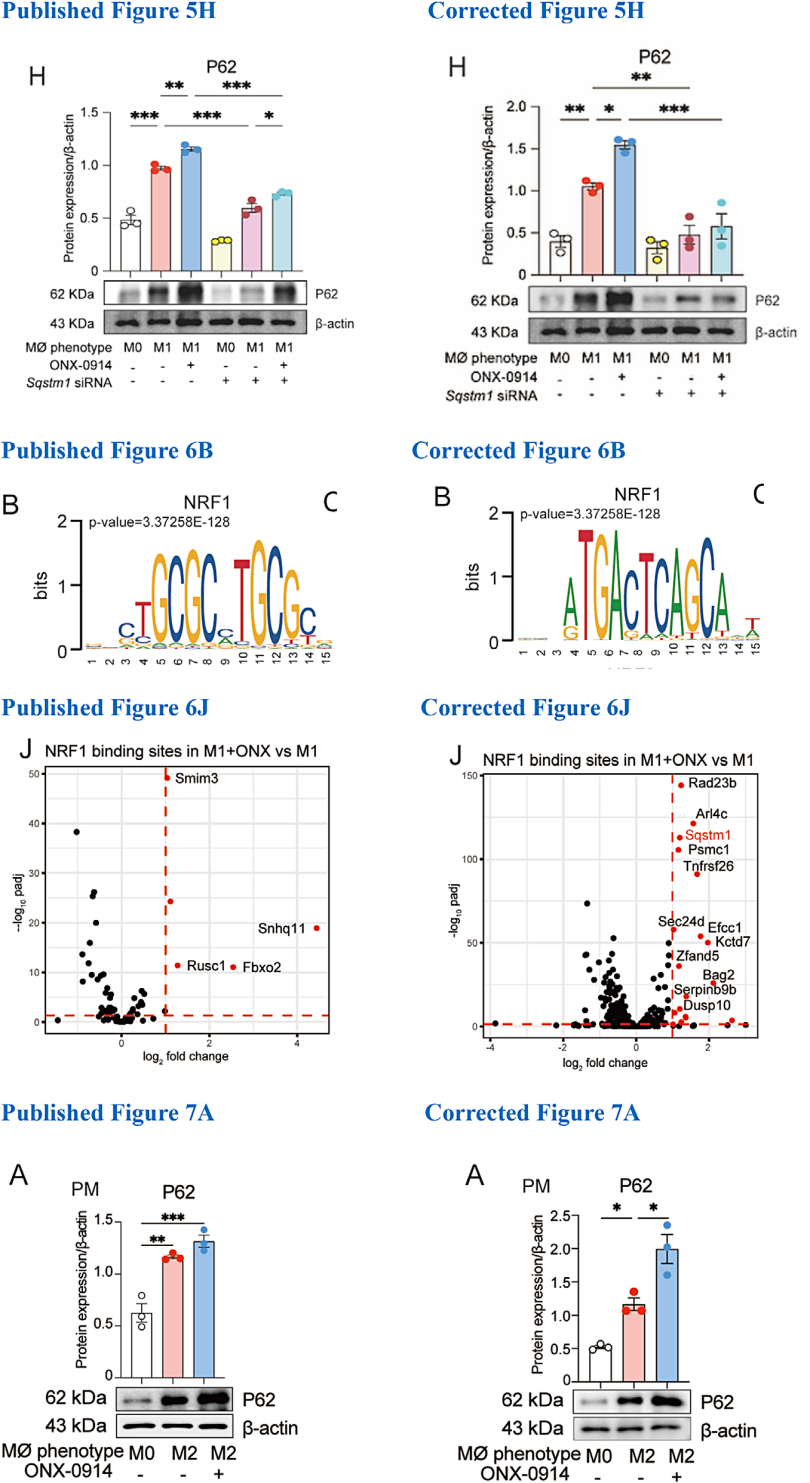


**TABLE 2:** Antibodies and reagents list of Western blotting and FACS.

Antibodies	Source	Identifier
anti‐rabbit ARG1 (1:1000)	Cell Signaling Technology	93668
anti‐rabbit Bip(1:1000)	Cell Signaling Technology	3177T
anti‐rabbit eIF2α (1:1000)	Cell Signaling Technology	5324T
anti‐rabbit p‐eIF2α (1:1000)	Cell Signaling Technology	3398T
anti‐rabbit IRF4 (1:1000)	Cell Signaling Technology	62834
anti‐rabbit GADD153 (CHOP) (1:1000)	Cell Signaling Technology	5554
anti‐rabbit IL‐1β (1:1000)	Cell Signaling Technology	31202
anti‐rabbit JNK1 (1:1000)	Cell Signaling Technology	3708T
anti‐rabbit iNOS (1:1000)	Cell Signaling Technology	2982
anti‐rabbit p‐JNK1 (1:1000)	Cell Signaling Technology	4668T
anti‐rabbit LMP2 (1:1000)	Abcam	ab242061
anti‐rabbit LMP7 (1:1000)	Abcam	Ab3327
anti‐rabbit LC3B (1:1000)	Cell Signaling Technology	2775S
anti‐rabbit TCF11/NRF1 (D5B10) (1:1000)	Cell Signaling Technology	8052
anti‐rabbit NRF2(1:500)	Cell Signaling Technology	12721T
anti‐rabbit STAT6 (1:1000)	Cell Signaling Technology	9362
anti‐rabbit p‐STAT6 (1:1000)	Cell Signaling Technology	56554
anti‐rabbit P62/SQSTM1 (1:1000)	Sigma‐Aldrich	P0067
anti‐rabbit YM1/2 (1:1000)	Abcam	ab192029
anti‐mouse β‐actin (1:3000)	Sigma‐Aldrich	A5316
HRP‐conjugated anti‐rabbit antibodies (1:3000)	Cell Signaling Technology	7074P2
HRP‐conjugated anti‐mouse antibodies (1:3000)	Cell Signaling Technology	7076S
7‐aminoactinomycin D (7‐AAD, 1:500)	Beyotime	ST515
Rat monoclonal anti‐mouse CD45 APC/Cyanine7 (1:300)	BioLengend	103116
Rat monoclona anti‐mouse CD170 (Siglec‐F) Brilliant Violet 421 (1:300)	BioLengend	155509
Rat monoclonal anti‐mouse/human CD11b AF700 (1:300)	BioLengend	101222
Hamster monoclonal anti‐mouse CD11c APC (1:300)	BioLengend	550261
Rat monoclonal anti‐mouse Ly‐6G FITC (1:300)	BioLengend	127605
Rat monoclonal anti‐mouse Ly‐6C PE/Cyanine7 (1:300)	BioLengend	128018

