# Supplementary material for: Targeting Immunoproteasome in Polarized Macrophages Ameliorates Experimental Emphysema Via Activating NRF1/2‐P62 Axis and Suppressing IRF4 Transcription
Source: Adv Sci (Weinh). 2024 Oct 2;11(44):2405318. doi: 10.1002/advs.202405318 (PMC11600198; doi:10.1002/advs.202405318)
Supplement: Supplementary file 1 — Supporting Information [file ADVS-11-2405318-s001.pdf]

ADVANCED SCIENCE Open Access

Supporting Information

for *Adv. Sci.*, DOI 10.1002/advs.202405318

Targeting Immunoproteasome in Polarized Macrophages Ameliorates Experimental Emphysema Via Activating NRF1/2-P62 Axis and Suppressing IRF4 Transcription

Bingxin Guo, Xing Shi, Qiong Jiang, Yuanwei Pan, Yuqiong Yang, Yuanyuan Liu, Shuyu Chen, Wenjiao Zhu, Laibin Ren, Ruifang Liang, Xue Chen, Haizhao Xu, Laiyou Wei, Yongjian Lin, Jinyong Wang, Chen Qiu, Haibo Zhou, Lang Rao, Lingwei Wang*, Rongchang Chen* and Shanze Chen**

Supporting Information

Targeting Immunoproteasome in Polarized Macrophages Ameliorates Experimental Emphysema *via* Activating NRF1/2-P62 Axis and Suppressing IRF4 Transcription

Bingxin Guo[#], Xing Shi[#], Qiong Jiang[#], Yuanwei Pan, Yuqiong Yang, Yuanyuan Liu, Shuyu Chen, Wenjiao Zhu, Laibin Ren, Ruifang Liang, Xue Chen, Haizhao Xu, Laiyou Wei, Yongjian Lin, Jinyong Wang, Chen Qiu, Haibo Zhou, Lang Rao, Lingwei Wang*, Rongchang Chen* and Shanze Chen**

Bingxin Guo, Xing Shi, Qiong Jiang, Yuqiong Yang, Shuyu Chen, Wenjiao Zhu, Laibin Ren, Ruifang Liang, Xue Chen, Haizhao Xu, Laiyou Wei, Yongjian Lin, Jinyong Wang, Chen Qiu, Lingwei Wang, Rongchang Chen and Shanze Chen
Department of Pulmonary and Critical Care Medicine, Shenzhen Institute of Respiratory Diseases
The First Affiliated Hospital (Shenzhen People's Hospital) and School of Medicine, Southern University of Science and Technology, Shenzhen 518055, China

Yuanyuan Liu, Ruifang Liang
Department of Respiratory Diseases and Critic Care Unit, Shenzhen Institute of Respiratory Disease, Shenzhen Key Laboratory of Respiratory Disease, Post-doctoral Scientific Research Station of Basic Medicine, The Second Clinical Medical College, Jinan University, Guangzhou 510632, China

Yuanwei Pan, Lang Rao
Institute of Chemical Biology, Shenzhen Bay Laboratory, Shenzhen 518132, China

Yuqiong Yang
National Clinical Research Center for Respiratory Disease, Guangzhou Institute of Respiratory Health, State Key Laboratory of Respiratory Disease, First Affiliated Hospital of Guangzhou Medical University, Guangzhou 510150, China

Yongjian Lin, Haibo Zhou
College of Pharmacy, Jinan University, Guangzhou, Guangdong 510632, China

[#] These authors contributed equally to this work.

*Corresponding e-mail: lrao@szbl.ac.cn; wang.lingwei@szhospital.com; chenrc@vip.163.com; chenshanze@mail.sustech.edu.cn.

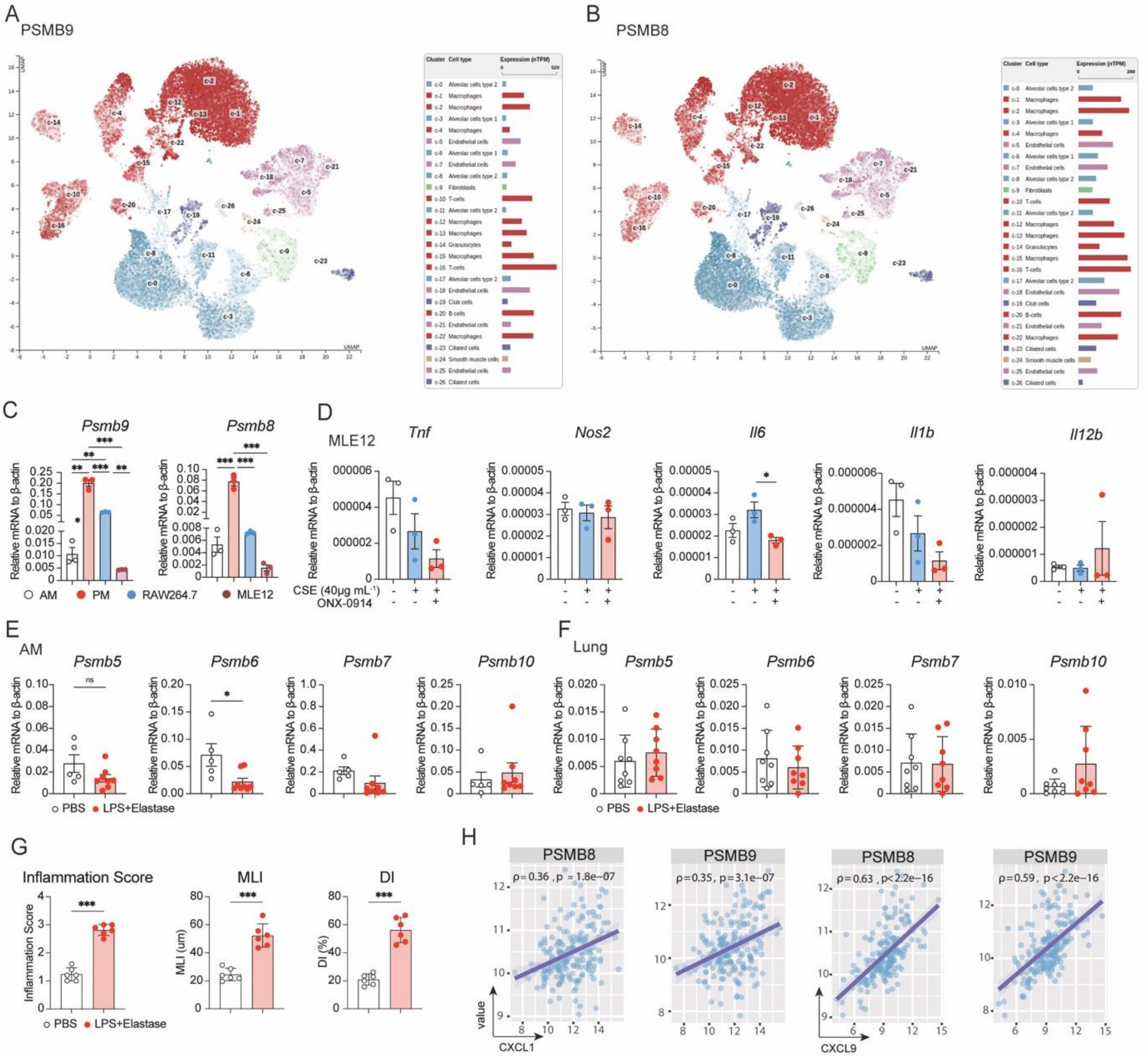

Figure S1. A and B) Publicly available scRNA-seq data of Human Protein Atlas (www.proteinatlas.org) was used to evaluate the expression of PSMB9 and PSMB8. C) RT-qPCR analyzed the expression of *Psmb9* and *Psmb8* in different macrophages (AM, PM, and RAW264.7 cell line) and alveolar epithelial cells (MEL12 cell line). D) After pretreated MEL12 cells with ONX-0914 for 6 h, followed by stimulation with 40 μg mL^{-1} CSE for 24 h, RT-qPCR examined M1 markers (*Nos2*, *Il12b*, *Il1b*, *Il6* and *Tnf*). E and F) RT-qPCR analysis detected *Psmb5*, *Psmb6*, *Psmb7* and *Psmb10* in AMs (E) and lung tissue (F) of each group. Data was collected from 8 independent samples. G) The inflammation and structural destruction of lung tissues were statistically analyzed by inflammation score, mean linear intercept (MLI) and destruction index (DI) in PBS and LPS + Elastase group (n = 7). H) The relationship between M1 marker genes (CXCL1 and CXCL9) and immunoproteasome-related genes (PSMB8, PSMB9) was reanalyzed using a clinical cohort of sputum transcriptomes from 99 COPD patients and 36 healthy individuals in China. *p < 0.05, **p < 0.01, ***p < 0.001.

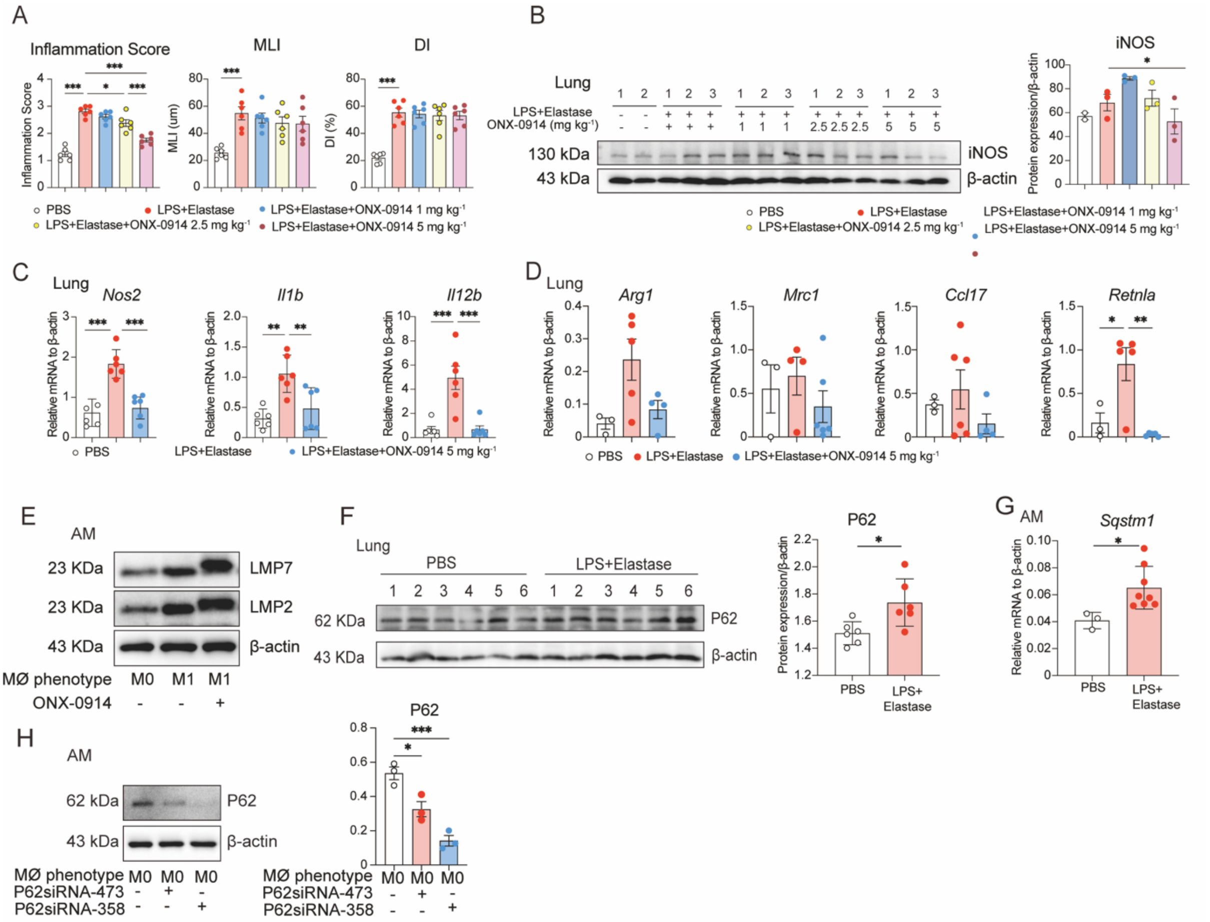

Figure S2. A) The inflammation and structural destruction of lung tissues from each group was statistically analyzed by inflammation score, MLI and DI. B) The expression levels of M1 marker protein iNOS were analyzed in lung tissue of each group by Western blotting analysis. C and D) The expression of M1 marker genes (*Nos2, Il1b, and Il12b*) and M2 marker genes (*Arg1*, *Ccl17, Mrc1*, and *Retnla*) were analyzed in lung tissue of each group by RT-qPCR. E) After pretreatment with 0.2 µM ONX-0914 for 6 h, LMP2 and LMP7 was analyzed by Western blotting in M1 polarization of AMs. F) The expression of P62 was analyzed in lung tissue of emphysema by Western blotting. G) RT-qPCR analysis detected *Sqstm1* expression in AMs of emphysema models. H) After 0.2 µM ONX-0914 treatment in AMs and silencing of P62 expression with siRNA-473 and siRNA-358, the expression of P62 was analyzed by Western blotting (n = 3). *$P < 0.05$, **$P < 0.01$, ***$P < 0.001$.

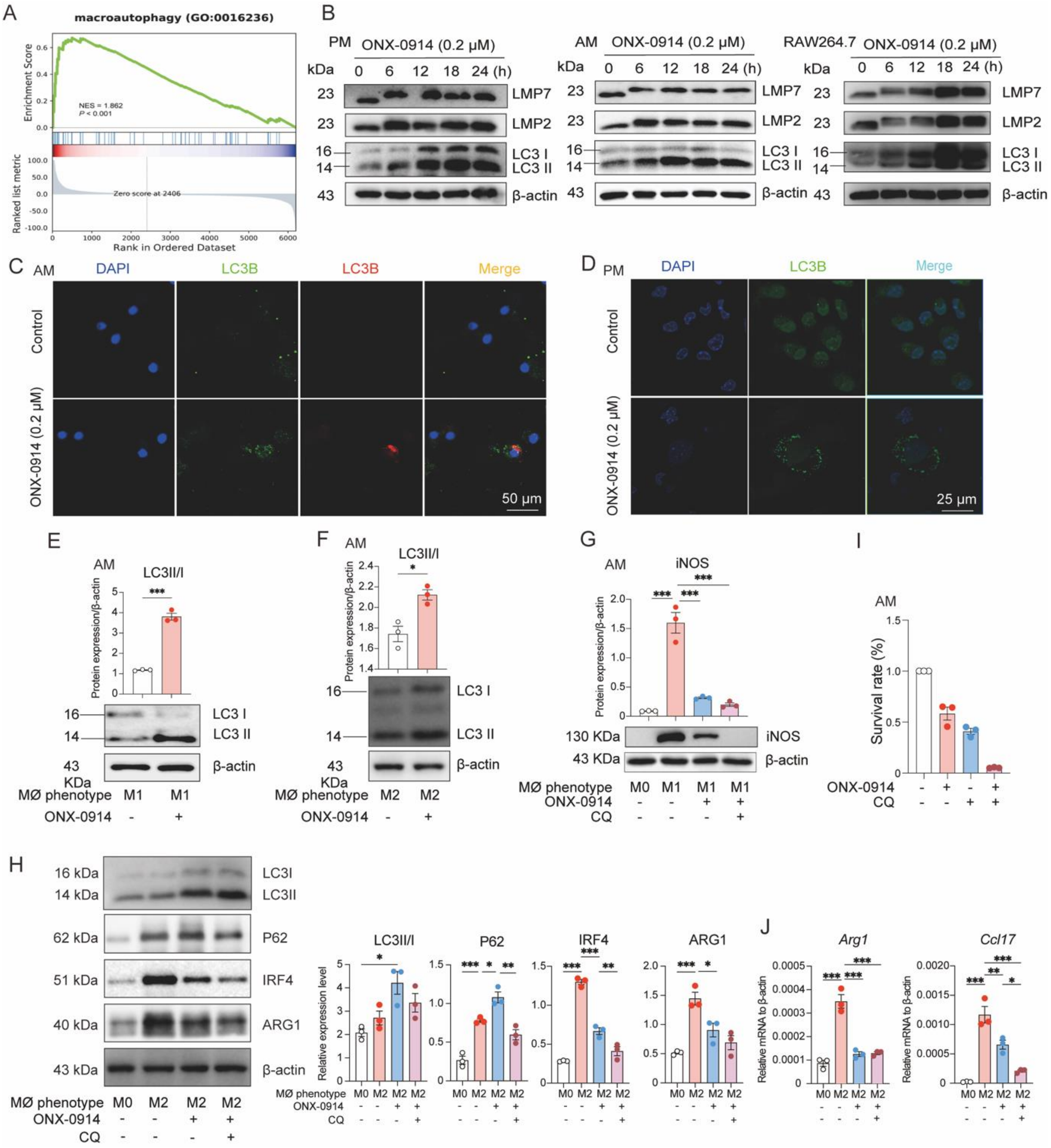

Figure S3. ONX-0914 Induced Autophagy and ERS Stress Activation was not Required for its Inhibitory Effect on M1 Polarization. A) GSEA for Differentially Expressed Genes (DEGs) of M1 after ONX-0914 treatment. The y-axis represents the enrichment score, and the x-axis is the ranked list of genes from highest to lowest based on statistical significance after ONX-0914 treatment. The results showed that macroautophagy was activated by ONX-0914 (*n* = 4). B) Representative Western blotting of immunoproteasome subunits LMP7, LMP2, and autophagy-related protein LC3II/I in PMs, AMs, and RAW264.7 cell line in response to 0.2 μM ONX-0914 treatment for 0, 6, 12, 18, and 24 h (*n* = 3). C) Applying 0.2 μM ONX-0914 to AMs for 24 h, the fluorescence intensity of LC3B was shown by the IF staining (*n* = 3). D)

Applying 0.2 μM ONX-0914 to PMs for 24 h, the fluorescence intensity of LC3B was shown by the double-immunofluorescence assay (*n* = 3). E) The ratio of LC3BII/I was analyzed in M1 phenotype of AMs with pretreatment of 0.2 μM ONX-0914 displayed by Western blotting analysis (*n* = 3). F) The ratio of LC3II/I was analyzed in M2 phenotype of AMs with pretreatment of 0.2 μM ONX-0914 displayed by Western blotting analysis (*n* = 3). G) The expression of M1 marker protein iNOS was detected by Western blotting in AMs with pretreatment of 0.2 μM ONX-0914 for 6 h followed by treating with 10 μM CQ and LPS / IFNγ-induced M1 AM for 24 h (*n* = 3). H) The expression of M2 marker protein, such as IRF4 and ARG1, and autophagy-related protein P62 were detected by Western blotting in PMs with pre-treatment of 0.2 μM ONX-0914 for 6 h followed by treating with 10 μM CQ and IL-4 induced M2 macrophage polarization for 24 h (*n* = 3). I) CCK8 assay evaluated cell viability of AMs that influenced by ONX-0914 and CQ (*n* = 3). J) M2 marker genes (*Arg1* and *Ccl17*) and autophagy-related gene *Sqstm1* that ONX-0914 and CQ effected were examined by RT-qPCR in AMs. *$P < 0.05$, **$P < 0.01$, ***$P < 0.001$.

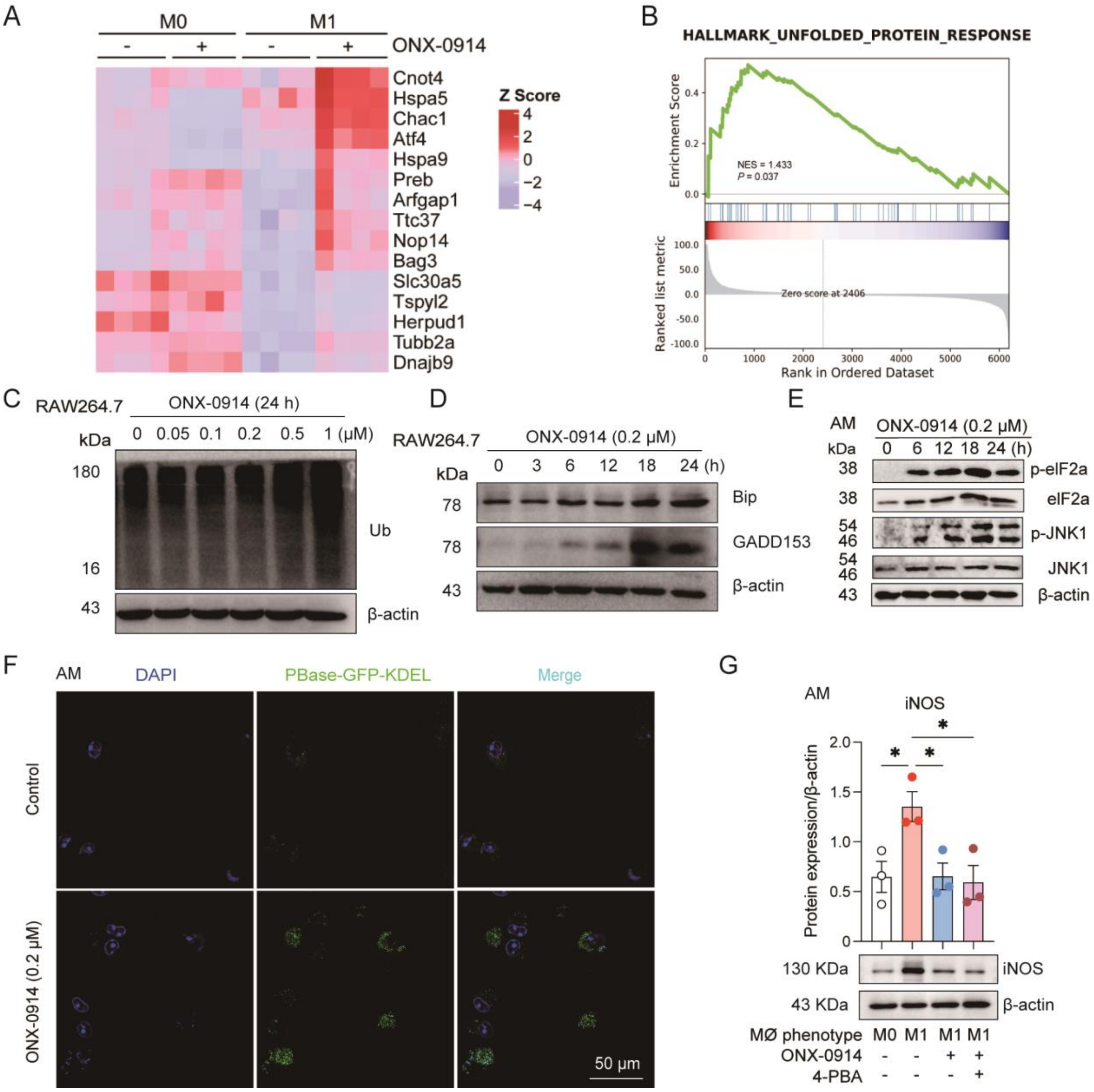

Figure S4. A) Heatmap depicting ERS-related genes that were significantly upregulated in 0.2 μM ONX-0914 pretreated M1 phenotype compared to other groups. B) GSEA for DEGs of M1 after ONX-0914 treatment. The results show that ERS was activated by ONX-0914 ($n = 4$). C) The expression of Ub were analyzed in RAW264.7 cell line in response to 0, 0.05, 0.1, 0.2, 0.5 μM dosage of ONX-0914 treatment for 24 h by Western blotting analysis (n = 3). D) The expression of Bip and GADD153 were analyzed in RAW264.7 cell line with 0.2 μM ONX-0914 treatment for 0, 3, 6, 12, 18, and 24 h by Western blotting analysis ($n = 3$). E) After 0.2 μM ONX-0914 treatment with different time courses, ERS-related proteins, including p-eIF2a, eIF2a, p-JNK1, JNK1, and Ub were analyzed by Western blotting analysis in AMs. F) Applying 0.2 μM ONX-0914 to AMs for 24 h, the fluorescence intensity of ER Stress was shown by the IF staining. G) The expression of iNOS was detected by Western blotting in AMs with

pretreatment of 0.2 μM ONX-0914 for 6 h followed by treating with 100 nM 4-Phenylbutyric acid (4-PBA) and LPS/IFNγ-induced M1 macrophage polarization for 24 h (n = 3). *$P < 0.05$, **$P < 0.01$, ***$P < 0.001$.

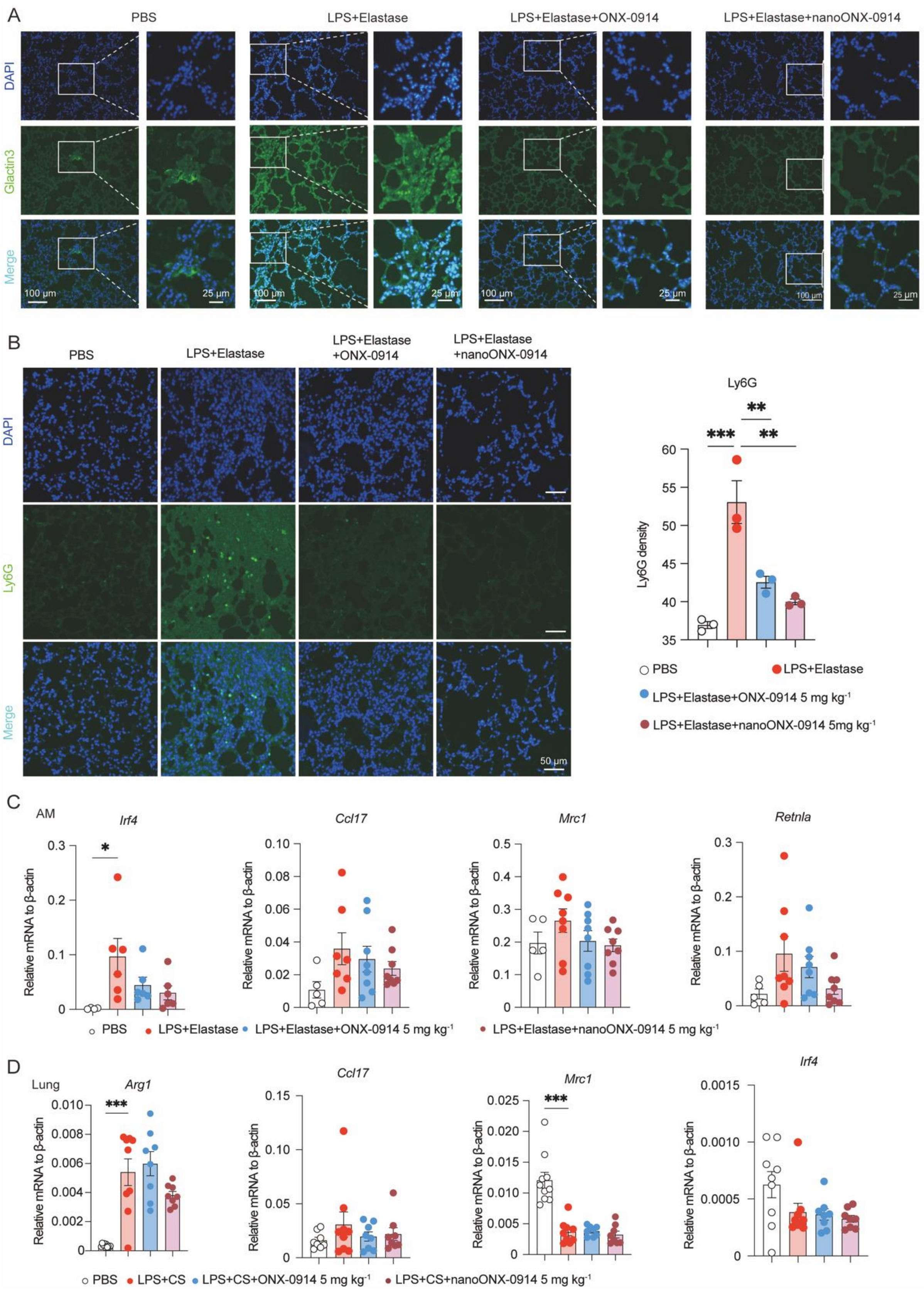

Figure S5. A) IF staining for Galectin-3 was used to assess the infiltration of macrophages in each group. B) Representative images and quantitation analysis of IF staining for Ly6G were used to assess neutrophil counts in lung tissue of each group. C) Expression analysis of M2

marker genes (*Irf4*, *Ccl17*, *Mrc1* and *Retnla*) in AMs of each group by RT-qPCR ($n \geq 5$). D) The expression of M2 marker genes (*Arg1*, *Ccl17, Mrc1*, and *Irf4*) were analyzed in lung tissue of each group by RT-qPCR. *$P < 0.05$, **$P < 0.01$, ***$P < 0.001$.

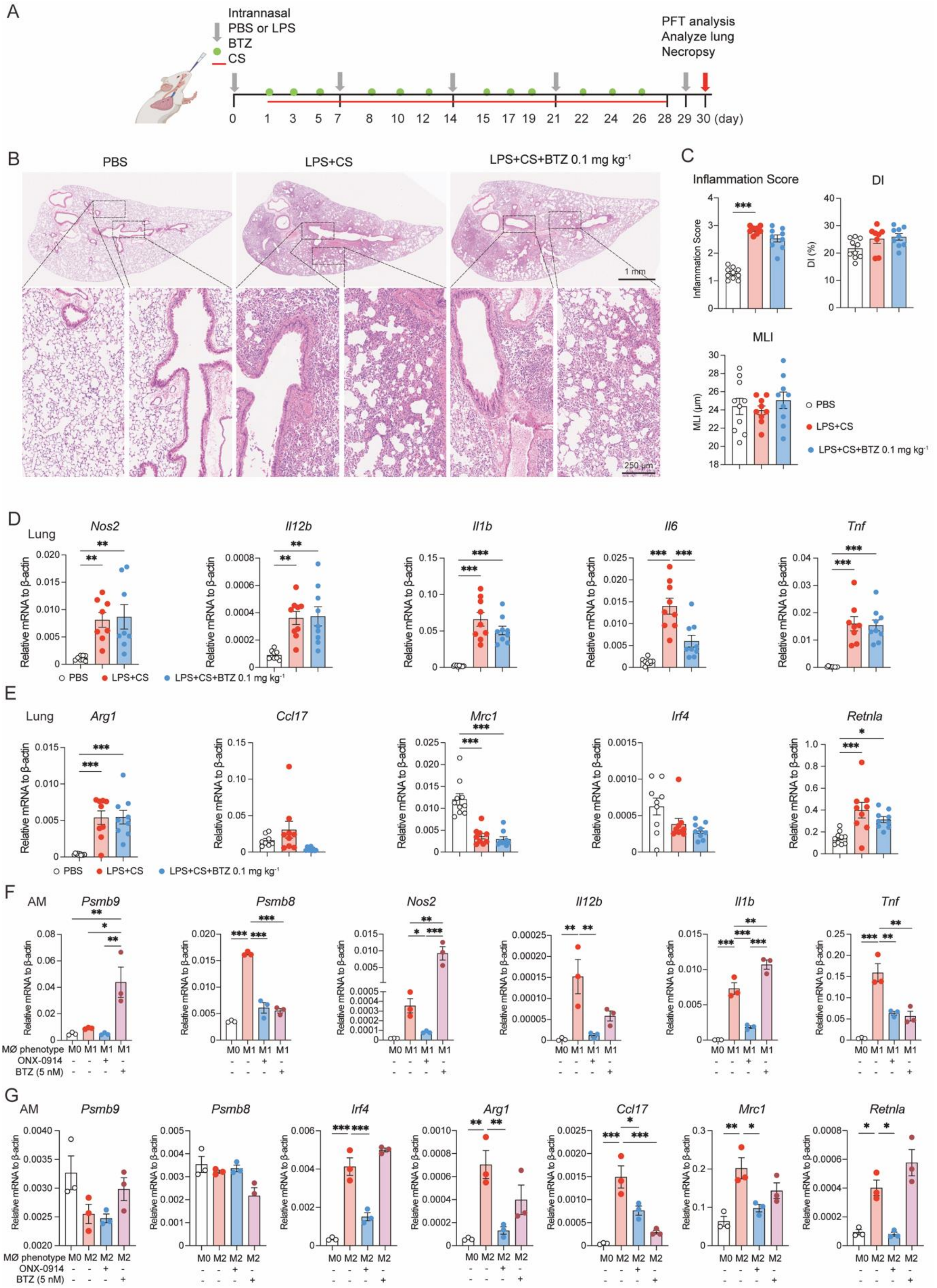

Figure S6. The Application of Proteasome Inhibitors BTZ in the Mouse LPS + CS model. A) I) Experimental setup for LPS + CS mouse models. BTZ was intranasally administered one day post-LPS challenge, three times weekly for four weeks to induce COPD and each group

contains at least 6 mice. B) H&E staining observed the alveolar changes in lung tissue in each group. C) The inflammation and structural destruction of lung tissues were statistically analyzed by inflammation score, MLI and DI. D and E) RT- qPCR analysis detected M1 marker genes (*Nos2*, *Il12b*, *Il1b*, *Il6* and *Tnf*) and M2 marker genes (*Arg1*, *Ccl17, Mrc1 Irf4*, and *Retnla*) in lung tissue of each group (n = 8). F) After pre-treatment with 0.2 μM ONX-0914 or 0.5 nM BTZ for 6 h, *Psmb9*, *Psmb8*, and M1 marker genes *Nos2*, *Il12b*, *Il1b*, and *Tnf* were examined by RT-qPCR in AMs (n = 3). G) After pretreatment with 0.2 μM ONX-0914 or 0.5 nM BTZ for 6 h, *Psmb9*, *Psmb8*, and M2 marker genes *Irf4*, *Arg1*, *Ccl17*, *Mrc1* and *Retnla* were examined by RT-qPCR in AMs (n = 3). *$P < 0.05$, **$P < 0.01$, ***$P < 0.001$.
